# Genome-wide *in vivo* CRISPR activation screen identifies BACE1 as a therapeutic vulnerability of lung cancer brain metastasis

**DOI:** 10.1126/scitranslmed.adu2459

**Published:** 2025-07-02

**Authors:** Shawn C. Chafe, Kui Zhai, Nikoo Aghaei, Petar Miletic, Zhi Huang, Kevin R. Brown, Daniel Mobilio, Daniel Young, Yujin Suk, Shan Grewal, Dillon McKenna, Zahra Alizada, Agata M. Kieliszek, Fred C. Lam, Laura Escudero, Qian Huang, Ariana Huebner, Jack Lu, Patrick Ang, Alisha Anand, Stefan Custers, Erika Apel, Sarah Slassi, Benjamin Brakel, Jongmyung Kim, James K. C. Liu, Blessing Iquo Bassey-Archibong, Rober Abdo, Yaron Shargall, Jian-Qiang Lu, Jean-Claude Cutz, Qi Zhang, Shawn Shun-Cheng Li, Chitra Venugopal, Robert E. Hynds, Antoine Dufour, Jason Moffat, Charles Swanton, Shideng Bao, Sheila K. Singh

**Affiliations:** 1 Department of Surgery, McMaster University; Hamilton, ON, Canada; 2 Centre for Discovery in Cancer Research, McMaster University; Hamilton, ON, Canada; 3 Department of Cancer Biology, Lerner Research Institute, Cleveland Clinic; Cleveland, OH, USA; 4 Department of Biochemistry and Biomedical Sciences, McMaster University; Hamilton, ON, Canada; 5 The Hospital for Sick Children; Toronto, ON, Canada; 6 Department of Physiology and Pharmacology, Cumming School of Medicine, University of Calgary; Calgary, Alberta, Canada; 7 Snyder Institute for Chronic Diseases, Hotchkiss Brain Institute and McCaig Institute for Bone and Joint Health, Cumming School of Medicine, University of Calgary; Calgary, AB, Canada; 8 Department of Neurosurgery, Stanford School of Medicine; Palo Alto, CA, USA; 9 Cancer Evolution and Genome Instability Laboratory, The Francis Crick Institute; London, England, UK; 10 Cancer Research UK Lung Cancer Centre of Excellence, UCL Cancer Institute, University College London, Department of Medical Oncology; London, England, UK; 11 Department of Radiation Oncology, Moffitt Cancer Center; Tampa, FL, USA; 12 Department of Neuro-oncology, Moffitt Cancer Center; Tampa, FL, USA; 13 Department of Biological Sciences, Concordia University of Edmonton; Edmonton, AB, Canada; 14 Department of Biochemistry, Schulich School of Medicine and Dentistry, Western University; London, ON, Canada; 15 Department of Pathology and Molecular Medicine, Western University; London, ON, Canada; 16 Division of Thoracic Surgery, McMaster University; Hamilton, ON, Canada; 17 Department of Pathology and Laboratory Medicine, McMaster University; Hamilton, ON, Canada; 18 Department of Pathology and Laboratory Medicine, London Health Sciences Centre, London, ON, Canada; 19 Epithelial Cell Biology in ENT Research Group, Developmental Biology and Cancer Department, UCL Great Ormond Street Institute of Child Health, University College London; London, UK; 20 Case Comprehensive Cancer Center, Case Western Reserve University School of Medicine; Cleveland, OH, USA; 21 Center for Cancer Stem Cell Research, Lerner Research Institute, Cleveland Clinic; Cleveland, OH, USA

## Abstract

Brain metastasis occurs in up to 40% of patients with non-small cell lung cancer (NSCLC). Considerable genomic heterogeneity exists between the primary lung tumor and respective brain metastasis; however, the identity of the genes capable of driving brain metastasis is incompletely understood. Here, we carried out an *in vivo* genome-wide CRISPR activation (CRISPRa) screen to identify molecular drivers of brain metastasis from an orthotopic NSCLC patient-derived xenograft model. We discovered activating expression of the Alzheimer’s disease associated β-site amyloid precursor protein cleaving enzyme 1 (BACE1) led to a significant increase in brain metastasis. Furthermore, genetic and pharmacological inhibition of BACE1 blocked NSCLC brain metastasis. Mechanistically, we identified BACE1 acts through its novel substrate EGFR to drive this metastatic phenotype. Together, our data highlights the power of *in vivo* CRISPR screening to unveil novel molecular drivers and potential therapeutic targets of NSCLC brain metastasis.

## Introduction

Brain metastasis is a uniformly fatal disease. Arising most commonly from primary tumors originating in the lung, breast and skin, brain metastases (BM) are the most common brain tumor in adults (1). It is estimated that approximately 20% of patients with solid tumors will eventually develop BM (2). Notably, incidence rates are on the rise, with BM currently matching those of breast and lung cancer (1). In the case of lung adenocarcinoma (LUAD), one of the most common sources of BM, 25% of patients will present with, and as many as 50% will eventually develop BM (3). Molecular alterations associated with increased likelihood of developing BM from LUAD include activating mutations in *KRAS* and *EGFR*, as well as *ALK* rearrangements (2). Survival times for these patients range from 4-15 months, and while molecular targeted therapeutics, including those targeting EGFR and KRAS, have delayed progression-free survival, treatment resistance often develops (4, 5). Thus, there is a critical need to improve therapeutic options for patients with BM.

Molecular profiling of BM has identified clonal divergence of the metastatic brain tumor from the primary tumor, revealing both the presence of mutations leading to therapeutic resistance as well as novel actionable mutations not present in the primary tumor (6), suggesting particular clones evolve with the capacity to seed BM. Further profiling studies have identified amplification of *MYC, YAP1* and *MMP13* are associated with increased development of LUAD-BM (7). While critical to our understanding of clonal evolution of BM, these analyses overlook the importance of non-mutated genes in the development of BM. To this end, *TWIST2* and *SPOCK1* have been shown to be important for supporting growth of non-small cell lung cancer (NSCLC) BM through loss-of-function screening in brain metastasis initiating cells (BMICs) (8). While analyses of transcriptional signatures associated with LUAD-BM have identified the importance of WNT/TCF (9), STAT3 (10), serpins (11) and HLA-G (12), these studies do not address whether expression of these genes is sufficient for driving dissemination from the lung to the brain. Here, we employed a genome-wide *in vivo* CRISPR activation screen to identify drivers of NSCLC BM from orthotopic LUAD tumors. In doing so, we identified the β-site amyloid precursor protein cleaving enzyme 1, also known as β-secretase 1, (*BACE1*) as an important regulator of NSCLC dissemination to and colonization of the brain. Mechanistically, we found that BACE1 acts through EGFR to promote metastatic phenotypes.

## Results

### *In vivo* CRISPR activation screen identifies BACE1 as a driver of lung adenocarcinoma brain metastasis

**Fig. 1:**
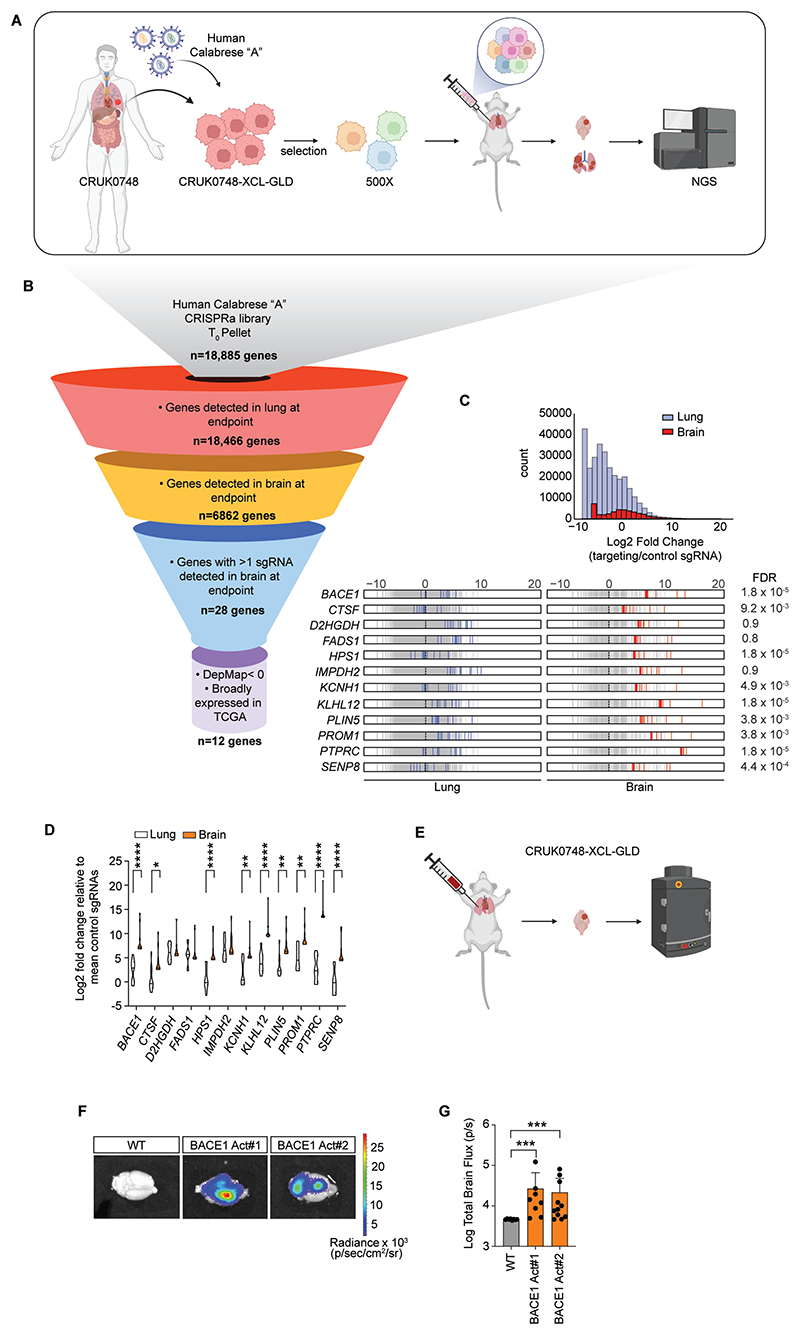
*In vivo* CRISPR activation screen identifies *BACE1* drives LUAD brain metastasis. (**A**) *In vivo* CRISPR activation screen schematic. NGS – next generation sequencing. (**B**) Schematic depicting our screen hit prioritization strategy. (**C**) Distribution and rug plots displaying log_2_ fold change of normalized sgRNA read counts (blue ticks – lungs, red ticks – brains) from individual lungs and brains relative to the mean of the control sgRNAs from the respective tissue. The distribution of the control sgRNAs is displayed as gray lines. Dotted line is log_2_ fold change of 0. (**D**) Violin plot depicting the relative enrichment of the sgRNAs of the indicated genes in the brain or lung as log_2_ FC compared to the control sgRNAs of the indicated tissue (*n*=12, one experiment). (**E**) Experimental scheme for orthotopic (intralung) implantation of CRISPR-activated *BACE1* CRUK0748-XCL-GLD cells. (**F**) *Ex vivo* bioluminescent images of brains from mice in the indicated groups at endpoint (*n*=10 per group, one experiment). (**G**) Quantification of the total flux of the *ex vivo* brain bioluminescent images in (F). Data in (C) were analyzed by one-sided Wilcoxon rank sum test with Benjamini-Holchberg correction (FDR). Data in (D) were analyzed by two-way ANOVA. Data in (G) were analyzed by Mann-Whitney U test. *P<0.05, **P<0.01, ***P<0.001, ****P<0.0001

To identify genetic drivers promoting the spread of LUAD to the brain in a clinically-relevant model, we made use of a primary LUAD patient-derived cell line CRUK0748-XCL, which originates from a patient-derived xenograft derived within the TRACERx study (Table S1) (13). We modified the cell line to express the catalytically inactive dCas9 (14) fused to VP64 (15) (fig. S1A), GFP and luciferase in order to activate gene transcription and to permit tracking of these cells *in vivo*, respectively and known from here onwards as CRUK0748-XCL-GLD cells. We confirmed the ability of dCas9 expression to induce target gene expression in cells *in vitro* by inducing CD45 expression following transduction of the CRUK0748-XCL-GLD cells with sgRNA to *PTPRC* (fig. S1B). We then proceeded to screen for genes whose expression would enhance BM from an orthotopic LUAD tumor. To do this, we utilized the human Calabrese genome-wide CRISPRa library (16). CRUK0748-XCL-GLD cells were transduced with the library at an MOI of 0.3 and selected to yield a final library coverage of 500x (Fig. 1A). We inoculated library-transduced cells directly into the lungs of 30 NSG mice using a modified thoracotomy procedure, providing 35x coverage of the library per mouse, as we have described previously in our established brain metastasis models (8, 10, 12, 17). We then followed tumor burden longitudinally by bioluminescent imaging. At endpoint, lungs and brains were collected and brains imaged by bioluminescent imaging to confirm the presence of metastases (fig. S1C). Genomic DNA extracted from both lungs and brains was sequenced to determine single guide RNA (sgRNA) abundance and identity. We started with sgRNAs targeting 18,885 genes present in our cell inoculum which was confirmed by sequencing of the T_0_ pellet (Fig. 1B). At endpoint we detected similar sgRNA representation in the lungs across all mice sequenced, suggesting that variability in engraftment rates would not impact our discovery approach (fig. S1D). Looking at the distribution of the sgRNA sequences in the lungs and the brains revealed an enrichment of a subset of gene targeting sgRNAs (fig. S1, E and F). Indeed, when we sequenced sgRNA sequences from the brains, we detected the presence of at least 1 sgRNA targeting 6862 unique genes. We then filtered our gene list by prioritizing genes with greater than one sgRNA present in the brains across all mice with an average abundance for the sgRNA in the brain cohort greater than in the lungs. Moreover, we also included genes with a single sgRNA enriched in the brain if the gene had 2 or fewer targeting sgRNAs detected in the lungs, which narrowed our list to 28 genes (Fig. 1B). Notably, since we expressed sgRNA as an enrichment in the brains relative to the lung this allowed us to select against genes involved in regulating proliferation where any enhancement in proliferation would be evident in both compartments. Indeed, none of the genes identified in our top 28 were known regulators of cell proliferation. Interrogating The Cancer Genome Atlas (TCGA) and DepMap (18) databases to prioritize genes broadly expressed in cancer and whose loss negatively affected cell viability across a panel of cancer cell lines, our list was further distilled to 12 genes which had an increased relative abundance of sgRNAs targeting them in the brain over the lung (Fig. 1, C and D, and Table S2). Among the genes in our shortlist was *CTSF*, the gene encoding cathepsin F. Cathepsin F was recently implicated as a biomarker of LUAD BM (19), adding independent biological validation to one of our hits and confirming the screen strategy was capable of identifying biologically relevant drivers of BM. Furthermore, cathepsin S, another member of the cathepsin family, has previously been shown to promote BM (20). We also implicated genes involved in lipid metabolism (*PLIN5, FADS1, D2HGDH*) which has been shown to be important for BM (21, 22). Moreover, genes previously identified in our BMIC gene signature as well as associated with brain tumor initiating cells (*IMPDH2, PROM1*) were also detected, independently confirming our previous findings and further supporting the validity of our strategy (23–25). In addition, we identified genes (*KLHL12, SENP8*) involved in regulating post-translational modifications of proteins, specifically CUL3, through ubiquitylation and NEDDylation, respectively, reinforcing the role of CUL3 in NSCLC (26, 27). Lastly, we identified sgRNA targeting *BACE1* were enriched greater than 150-fold in the brains of the mice in our cohort (Fig. 1D). Importantly, *BACE1* expression was elevated in our BMIC signature derived from disseminated lung cancer cells in the brain (12). Furthermore, we recently identified BACE1 was important for maintaining a tumor-promoting macrophage phenotype in glioblastoma, suggesting it might have a multi-faceted role in the progression of solid cancers in the brain (28). BACE1 is primarily known for its role in Alzheimer’s disease (AD) (29). It is a single-pass transmembrane protein member of the aspartyl protease family that is required for the production of amyloid-β (Aβ) peptide, which accumulates in the brains of patients with AD (29). Since *BACE1* has never been previously implicated in LUAD or BM, we decided to investigate its role in greater depth.

To confirm elevated *BACE1* expression in primary LUAD increased BM, we generated CRUK0748-XCL-GLD cells expressing the top two most efficacious *BACE1-*targeting sgRNAs from the screen (fig. S1G). We inoculated these cells directly into the lungs of NSG mice and followed tumor burden over time by bioluminescent imaging (Fig. 1E). As mouse endpoint in this model is dictated by primary lung tumor burden, we extracted brains at endpoint and imaged them by *ex vivo* bioluminescence imaging to detect metastatic brain signal (Fig. 1F). Consistent with the high ranking of *BACE1* in the screen, and in support of our primary screen findings, activation of its expression with two independent sgRNAs increased metastatic brain tumor burden (Fig. 1, F and G). Furthermore, we interrogated liver and bone for the presence of metastases to determine whether BACE1 expression could also enhance the spread of LUAD cells to these distant sites. Indeed, we observed increased metastatic burden in the livers as well as in the bones of mice with BACE1 over-expressing lung tumours (fig. S1, H and I). Together, these data provide additional validation for our pooled CRISPRa screen finding that increased expression of *BACE1* enhances LUAD BM while also providing evidence that BACE1 expression may predict LUAD metastasis.

### *BACE1* is expressed in LUAD brain metastases and is associated with shorter survival

**Fig. 2:**
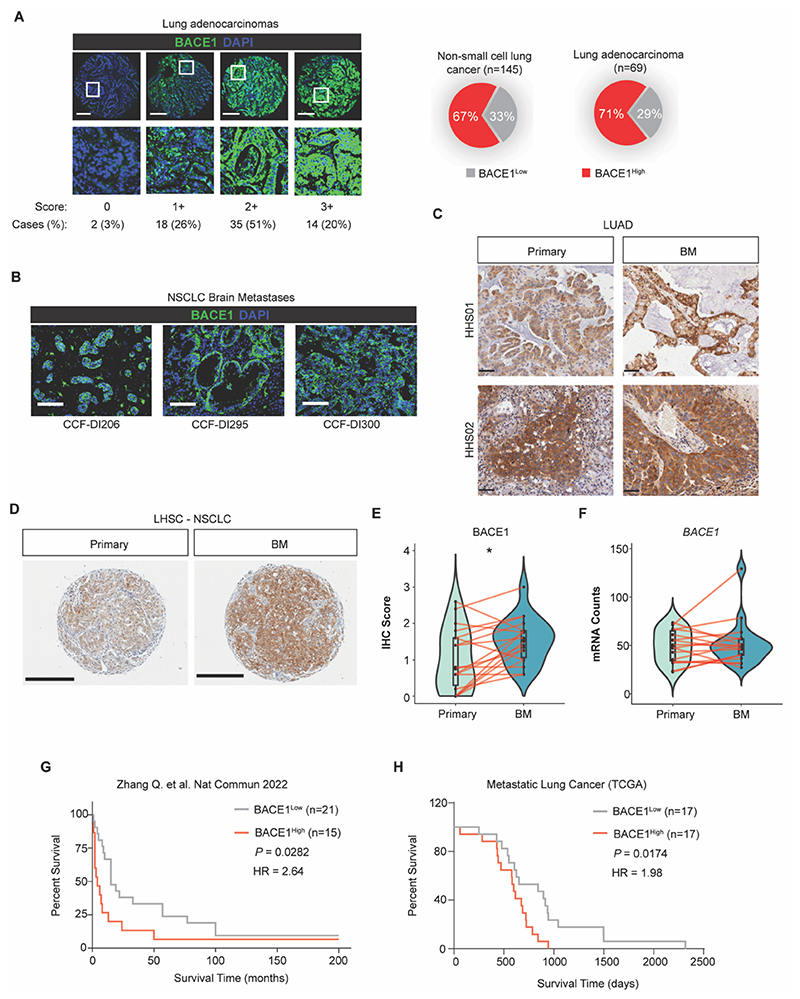
BACE1 is expressed in LUAD brain metastasis and is associated with worse prognosis. (**A**) (*Left*) Lung cancer tumor microarray (*n*=145) including lung adenocarcinomas (*n*=69) stained for BACE1. Green – BACE1, Blue – DAPI (nuclei). (*Right*) Pie charts indicating the proportion of tumors staining high or low for BACE1 in all non-small cell lung cancers (left) or in lung adenocarcinomas (right). Scale bar = 250 μm. (**B**) Immunohistochemical staining of brain metastases from patients with LUAD for BACE1. Colored as in (A). Scale bar = 250 μm. (**C**) Immunohistochemical staining of BACE1 in primary LUAD and matched brain metastases from Hamilton Health Sciences. Scale bar = 50 μm. (**D**) Immunohistochemical staining of BACE1 in the primary NSCLC and matched brain metastases TMA from London Health Sciences Centre (LHSC). Scale bar = 300 µm. **(E)** Violin plot depicting quantitation of BACE1 staining in (D) (*n*=21). **(F)** Violin plot depicting mRNA counts from GeoMx analyses of the samples in (D). **(G)** Kaplan-Meier curve depicting survival proportions for patients from the LHSC cohort according to BACE1 expression. **(H)** Kaplan-Meier curve depicting survival proportions for patients from the TCGA with LUAD brain metastases stratified according to median *BACE1* expression. Hazard ratio determined using multivariate analysis with Cox Proportional Hazards mode (G,H). Data in (E) was analyzed by t test. Data in (H) was analyzed by Log-rank test. *P<0.05

To evaluate whether BACE1 expression is clinically relevant in NSCLC, we stained a NSCLC tumor microarray (TMA) consisting of primary LUAD (Fig. 2A) and lung squamous cell carcinomas (LUSC; fig. S2, A and B) for BACE1. We found that BACE1 was highly expressed in 71% of all cores present in the TMA, suggesting BACE1 might be a biologically relevant molecule in NSCLC. Furthermore, we looked into the TRACERx 421 cohort for associations of *BACE1* expression with clonal driver mutations linked to the development of BM (fig. S2C). We found that *BACE1* expression was highest in *EGFR* mutant LUAD. We next assessed whether expression of BACE1 was also present in BM from patients with LUAD. We found that BACE1 was expressed in all 13 metastatic brain tumors interrogated (Fig. 2B, fig. S2D, and Table S3), but not in adjacent normal areas or most normal tissues outside of the brain (fig. S2, E and F). Additionally, we possessed two matched primary LUAD-BM pairs in our biobank that we were able to interrogate and assess whether BACE1 expression was preserved from primary to metastatic brain tumor (Fig. 2C, and Table S3). Notably, BACE1 expression in the primary tumor was maintained in the BM. Moreover, when we evaluated BACE1 expression in NSCLC tumors from patients that did not develop BM, BACE1 staining was minimal (fig. S2G). We then looked to extend these observations to an additional cohort of BM patients and stained a TMA consisting of 44 matched primary NSCLC and their matched BM for BACE1 (Fig. 2D) (30). BACE1 staining was detected in all but 3 primary lung tumours, yet was detected in the BM of all patients (Fig. 2E). Interestingly, the expression of *BACE1* at the RNA level in these cores was quite stable between the primary and BM, suggesting that BACE1 expression may be regulated post-translationally in LUAD (Fig. 2F). We then assessed whether *BACE1* expression in BM was associated with patient survival. Stratifying patients according to BACE1 expression in the London Health Sciences cohort revealed that those patients with high BACE1 expression in their BM survived for much shorter times from their BM diagnosis than those with lower BACE1 expression (Fig. 2G). We next interrogated the LUAD TCGA data where the metastatic status was available and found information for 34 patients with BM. When we stratified this patient cohort according to median expression of *BACE1*, those with high *BACE1* expression survived for a shorter duration than those with low *BACE1* expression (Fig. 2H). Moreover, we ran a multivariate analysis cox proportional hazards model with age, sex, stage and smoking status as co-variates and determined that high BACE1 expression was indeed associated with worse outcomes in both cohorts (HR^LHSC^ = 2.64, p=0.008; HR^TCGA^ = 1.98, p=0.004). Together, these data suggest that BACE1 is a clinically relevant target in NSCLC, expressed in both primary lung tumors and NSCLC brain metastases (LBM) where its expression is associated with worse prognosis.

### BACE1 drives migration and invasion of primary LUAD

**Fig. 3:**
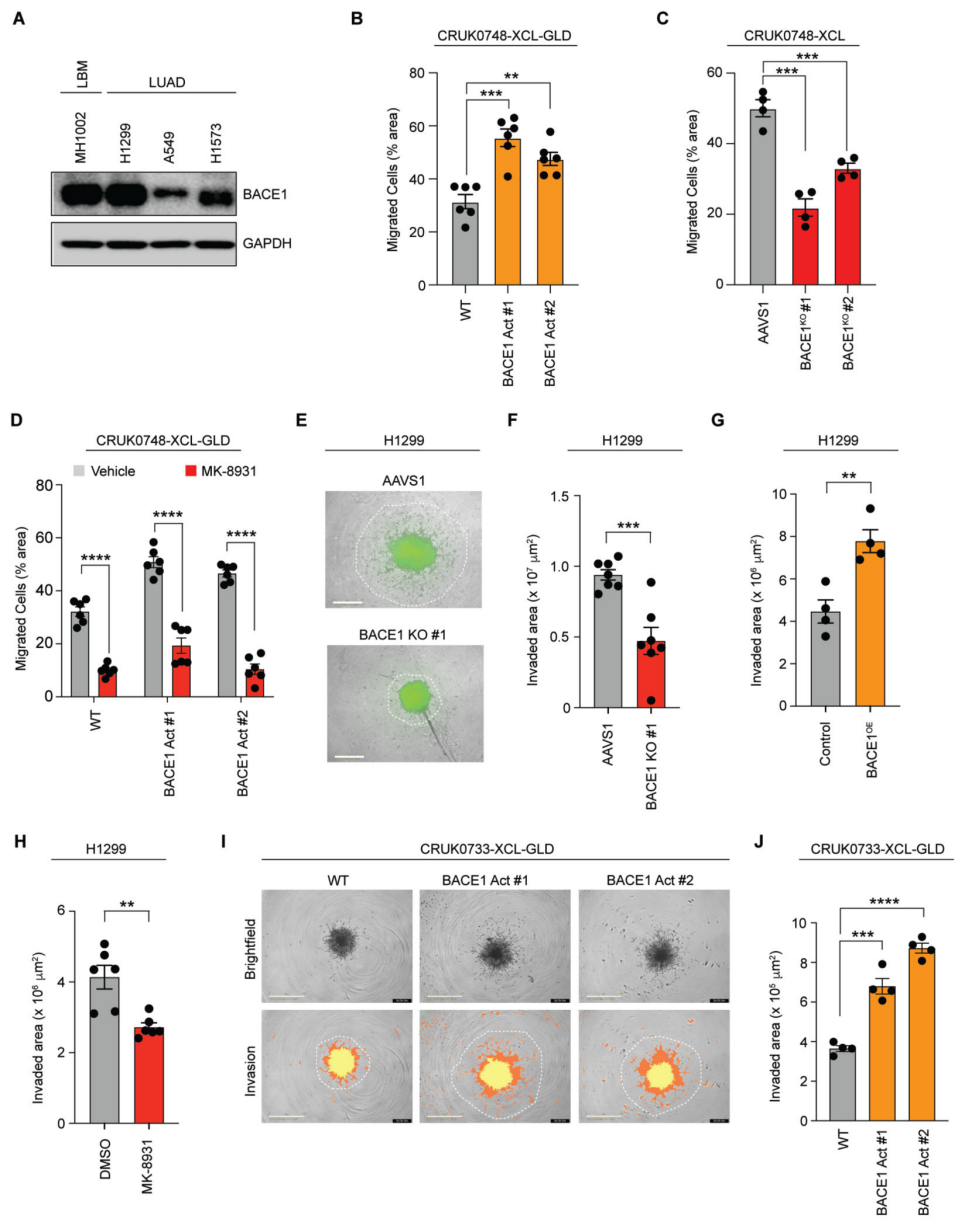
BACE1 increases the migratory and invasive capacity of primary LUAD cells. (**A**) Western blot analysis of BACE1 expression in a panel of non-small cell lung cancer and lung-to-brain metastasis (LBM) lines. (**B**) Quantification of the migrated cell area of CRUK0748-XCL-GLD cells following BACE1 activation (*n*=6, *N*=3). (**C**) Quantification of the migrated cell area of *BACE1*^KO^ CRUK0748-XCL cells (*n*=4, *N*=3). (**D**) Quantification of migrated cell area of MK-8931 (10 μM) treated CRUK0748-XCL-GLD cells (*n*=6, *N*=3). (**E**) Representative micrographs depicting spheroid invasion of BACE1^KO^ H1299^GFP^ after 7 days in Matrigel^™^. White boundaries indicate extent of invasion for the indicated cell lines. Scale bar = 800 μm. (**F**) Quantification of the invaded area of the indicated cell lines in (E) quantified using ImageJ (*n*=7, *N*=2). (**G**) Quantification of H1299^GFP^ invasion in a spheroid invasion model following overexpression of *BACE1* (*n*=4, *N*=2). (**H**) Quantification of H1299^GFP^ invasion in a spheroid invasion model following treatment with MK-8931 (50 μM) for 7 days (*n*=6, *N*=2). (**I**) Representative micrographs depicting spheroid invasion of CRISPR-activated *BACE1* CRUK0733-XCL-GLD cells after 80 hours in Matrigel^™^. Invasion images illustrate the quantification mask from the Incucyte® spheroid software module utilized to quantify the extent of invasion. Yellow marks the growth of the sphere; orange and white mark the extent of invasion. Scale bar = 1 mm. (**J**) Quantification of invasion in (I) (*n*=4, *N*=2). Bars represent the mean number of migrated cells or invaded area + SEM. Data in (B), (C), (F), (G), (H) and (J) was analyzed by t test. Data in (D) was analyzed by two-way ANOVA with Sidak’s multiple comparisons test. **P<0.01, ***P<0.001****P<0.0001.

To evaluate the biological relevance of BACE1 expression in LUAD and LBM, we assessed BACE1 expression in a panel of LUAD and patient-derived LBM cell lines in our biobank to adopt suitable models for further investigation (Fig. 3A and fig. S3A). To determine whether BACE1 expression supported aggressive phenotypes, we first evaluated whether BACE1-expressing cells were more migratory. Indeed, activation of BACE1 enhanced migration of CRUK0748-XCL-GLD cells through transwell membranes (Fig. 3B, and fig. S3B). In order to assess whether BACE1 expression was necessary for LUAD migration, we genetically deleted *BACE1* using CRISPR/Cas9 in CRUK0748-XCL cells (fig. S3C). In contrast to increased expression of BACE1, suppressing BACE1 expression reduced the migratory capacity of these cells (Fig. 3C, and fig. S3D). We next investigated whether BACE1 activity was required for the increased migration associated with BACE1 expression. To address this question, we made use of Verubecestat (MK-8931), a potent, blood-brain barrier permeable, small molecule inhibitor targeting BACE1 that has been trialed in AD (29, 31). Indeed, the increased migration associated with higher BACE1 expression was dependent upon BACE1 activity as MK-8931 reduced the ability of CRUK0748-XCL cells to migrate (Fig. 3D, and fig. S3E).

We next evaluated whether BACE1 expressing cells were more invasive using a spheroid invasion assay. We knocked BACE1 expression out in H1299 NSCLC cells and assessed their capacity to invade through Matrigel^™^ (fig. S3F). Suppression of BACE1 expression attenuated the invasive capacity of these cells (Fig. 3, E and F). Conversely, over-expression of BACE1 enhanced their invasive capacity (Fig. 3G, and fig. S3G). Moreover, this invasive phenotype was dependent upon BACE1 activity, as MK-8931 impeded their ability to invade (Fig. 3H). However, to truly assess the role of BACE1 in invasion, we made use of a non-metastatic patient-derived primary LUAD line, CRUK0733-XCL, and assessed whether increased BACE1 expression was sufficient to drive invasion (fig. S3H). Increased BACE1 expression in CRUK0733-XCL led to an increase in the invasive capacity of these cells (Fig. 3, I and J, Movies S1 and S2). Together these data support a cell-autonomous role for BACE1 in enhancing the invasive phenotype of NSCLC cells.

### BACE1 is required for the proliferation and self-renewal capacity of LUAD brain metastasis initiating cells

**Fig. 4:**
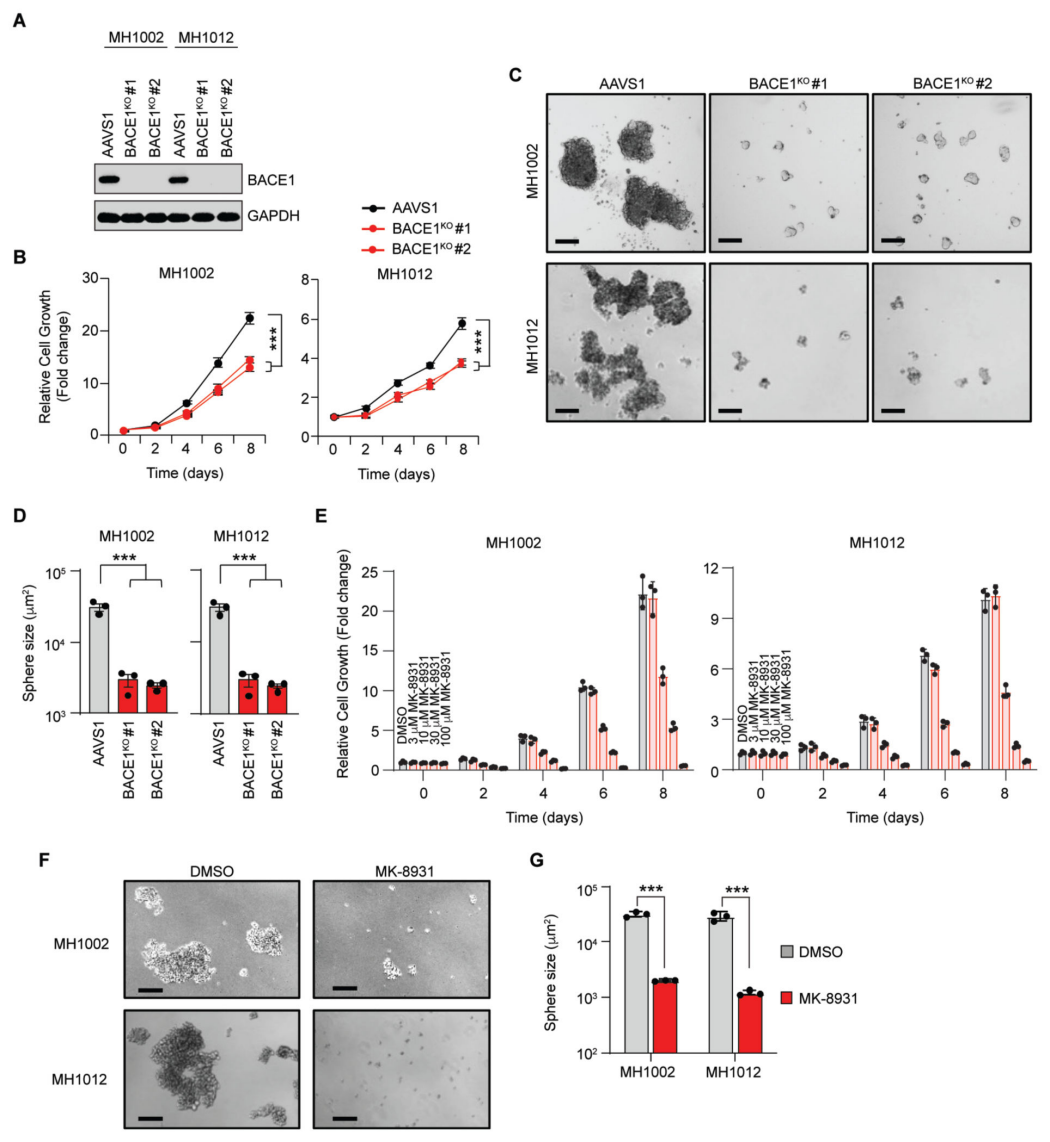
BACE1 is required for the proliferation and self-renewal capacity of LUAD brain metastases. (**A**) Western blot analysis of BACE1 expression in control (AAVS1^KO^) or BACE1^KO^ MH1002 cells. (**B**) Proliferation of BACE1^KO^ MH1002 (left) and MH1012 (right) cells (*n*=3, *N*=3). (**C**) Micrographs depicting the sphere formation capacity of BACE1^KO^ MH1002 (Top) and MH1012 (Bottom) cells. Scale bar = 200 μm. (**D**) Quantification of sphere size for the indicated cell lines in (C) (*n*=3, *N*=3). (**E**) Cell proliferation of the indicated cell lines in response to MK-8931 at the indicated doses after 72 hours (*n*=3, *N*=3). (**F**) Micrographs depicting the sphere formation capacity of the indicated cell lines in response to 50 μM MK-8931 treatment for 96 hours. Spheres were allowed to form for seven days (*n*=3, *N*=3). Scale bar = 200 μm. (**G**) Quantification of sphere size from the images in (F). Bars indicate mean + SEM. Data in (B) were analyzed by two-way ANOVA with Tukey’s multiple comparisons test. Data in (D) and (G) were analyzed by t test. ***p<0.001.

Since BACE1 supported phenotypes associated with LUAD dissemination, we next wanted to assess the role of BACE1 in brain metastatic LUAD cells. To assess whether BACE1 expression was important for LBM growth, we disrupted *BACE1* using CRISPR/Cas9 in the patient-derived MH1002 and MH1012 LBM lines (Fig. 4A, and Table S1) and assessed cell proliferation (Fig. 4B). Loss of BACE1 expression reduced LBM cell proliferation in both cell lines (Fig. 4B). We next grew MH1002 and MH1012 in stem cell enriching conditions to assess whether BACE1 was important for LBM self-renewal, a cardinal stem cell property (Fig. 4C). Similar to what we observed in our NSCLC models, loss of BACE1 expression reduced sphere forming capacity and sphere size in both LBM lines (Fig. 4, C and D, fig. S4A). Together, these findings suggest that BACE1 expression is important for LBM growth and self-renewal capacity.

### BACE1 enzymatic activity is important for the growth and self-renewal of LUAD LBM cells

We next assessed whether BACE1 enzymatic activity was also important for LBM proliferation and self-renewal capacity. We therefore tested the effect of MK-8931 on the growth of our patient-derived LBM cells. MK-8931 treatment of MH1002, MH1012, BT530 and BT478 reduced cell proliferation in a target-specific and dose-dependent manner (Fig. 4E, and fig. S4B and C). Moreover, we confirmed these findings with three additional BACE1 inhibitors, AZD3293, AZD3839, and PF-06751979 in MH1002 cells (fig. S4D). We next investigated whether BACE1 activity supported the self-renewal capacity of MH1002 and MH1012 cells (Fig. 4F). Growth of MH1002 and MH1012 in stem cell enriching conditions in the presence of MK-8931 reduced their ability to form spheres (Fig. 4G). Moreover, we made similar observations in CRUK0748-XCL and H1299 NSCLC cells (fig. S4, E to H). Collectively, these data confirm our genetic findings and suggest that BACE1 activity is important for sustaining the growth and self-renewal of LBMs, properties critical for supporting initiation and maintaining growth of disseminated cells in the brain.

### BACE1 is required for LUAD brain metastasis

**Fig. 5:**
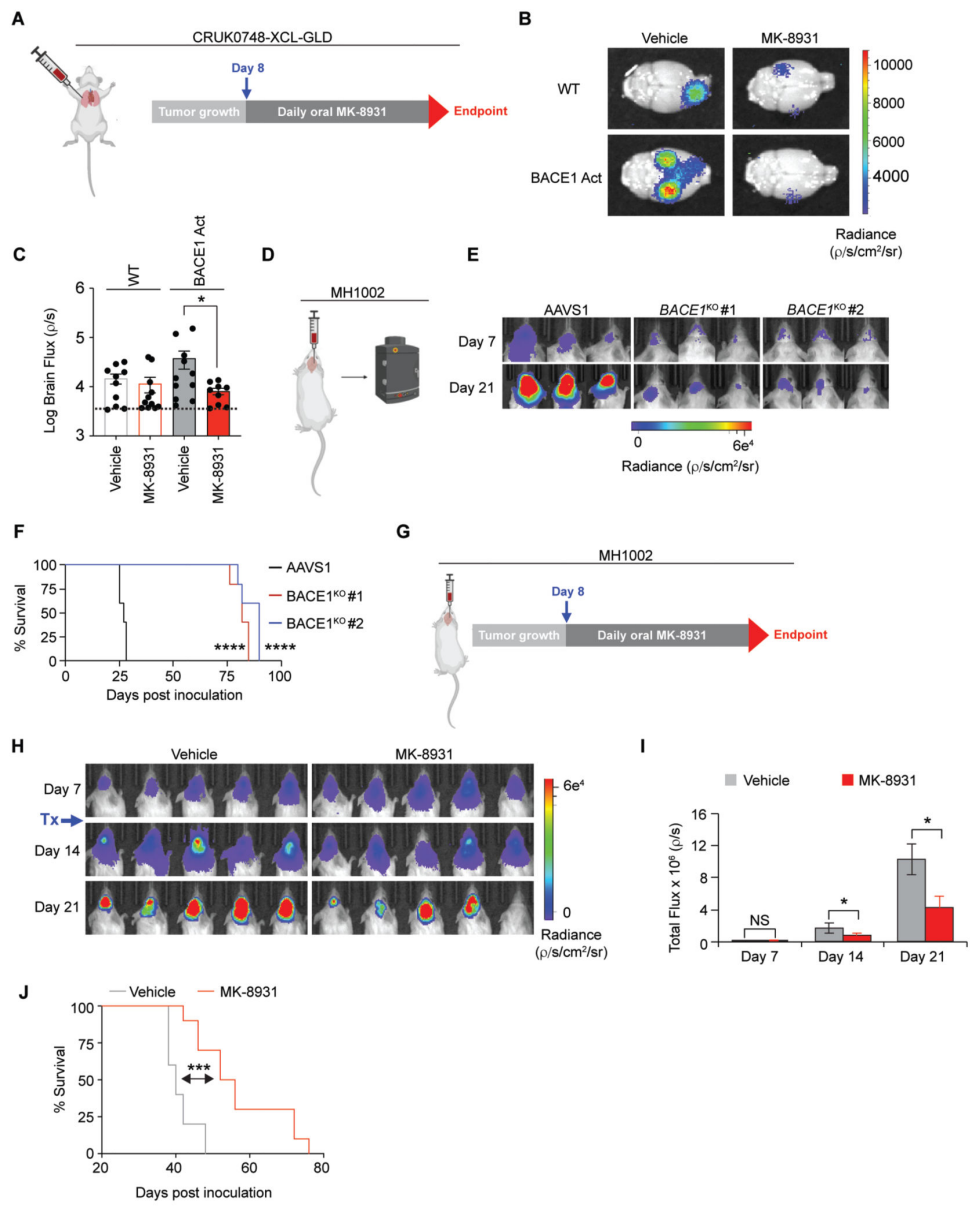
Perturbing BACE1 expression or activity blocks the formation of LUAD brain metastases. (**A**) Experimental scheme for orthotopic injection (intralung) of CRISPR-activated *BACE1* CRUK0748-XCL-GLD cells treated daily by oral gavage with 30 mg/kg MK-8931 for 21 days. (**B**) Representative *ex vivo* bioluminescent images of brains from the indicated groups. (**C**) Quantification of the total brain flux for all mice of the indicated groups. Dashed line indicates baseline luminescence (*n*=9-11 per group, one experiment). BACE1-Act Vehicle vs WT Vehicle *P*= 0.23. (**D**) Experimental scheme for intracranial injection of BACE1^KO^ MH1002 cells. (**E**) Longitudinal bioluminescent images of representative mice from the indicated groups (*n*=5 per group, one experiment). (**F**) Kaplan-Meier curve depicting the survival times of mice across the indicated groups. (**G**) Experimental scheme for intracranial injection of MH1002 cells treated daily by oral gavage with 30 mg/kg MK-8931 for 21 days. (**H**) Longitudinal bioluminescent images of representative mice from the indicated groups. (**I**) Quantification of the total brain bioluminescent flux for the indicated groups over time (*n*=8 per group, one experiment). (**J**) Kaplan-Meier curve depicting the survival times of the mice across the indicated groups. Bars indicate mean + SEM. Data in (C) were analyzed by Mann-Whitney U test. Data in (F) and (J) were analyzed by log-rank test. Data in (I) were analyzed by t test. *P<0.05, ***p<0.001, ****P<0.0001.

After demonstrating that genetically and pharmacologically perturbing BACE1 expression and activity, respectively, altered the growth and invasiveness of NSCLC and LBM lines *in vitro*, we wanted to assess whether we could limit the spread of BACE1-expressing disseminated LUAD cells to the brain by targeting BACE1 activity with MK-8931. To do this, we made use of our CRUK0748-XCL primary LUAD model expressing *BACE1*-activating sgRNA, and implanted these cells directly into the lungs of NSG mice (Fig. 5A). We allowed tumors to form for seven days before randomly assigning *BACE1*-activated, or control, tumor bearing mice to vehicle or MK-8931 treatment groups. Mice were treated daily for three weeks and then all mice were culled to assess any differential in metastatic brain tumor burden (Fig. 5B). Bioluminescence imaging of brains collected at endpoint confirmed increased expression of BACE1 enhanced the metastatic propensity of CRUK0748-XCL-GLD cells (Fig. 5, B and C). Moreover, MK-8931 treatment reduced the spread of this primary LUAD model to the brain (Fig. 5, B and C). Notably, MK-8931 had minimal impact on the wild-type cells which are much more inefficient at reaching the brain and express lower amounts of BACE1 (fig. S1G). Together, these data support a critical role for BACE1 activity in all stages of the development of LUAD BM.

### BACE1 expression is required for growth of established LBM

BM patients often present clinically with symptomatic lesions. To test whether we could target BACE1 in a setting of advanced disease, we used our orthotopic, intracranial implantation BM model. Given the link between self-renewal capacity *in vitro* and tumor initiating capacity *in vivo* (32), we first assessed whether BACE1 expression was important for LBM tumor growth in the brain. To test this, we inoculated MH1002 *BACE1*^KO^ or control (*AAVS1*^KO^) cells into the brains of immunocompromised mice and monitored tumor growth (Fig. 5D). We found that loss of BACE1 expression markedly impaired LBM tumor growth in the brain, which resulted in a considerable extension in survival (Fig. 5, E and F). Since BACE1 expression was required for sustaining LBM growth in the brain, we next assessed whether MK-8931 would also impact the growth of LBM in the brain. MH1002 cells were inoculated into the brain of NSG mice, allowed to form tumors for seven days, after which time MK-8931 was administered daily for three weeks (Fig. 5G). Treatment with MK-8931 reduced LBM brain tumor growth, which led to a marked extension in survival (Fig. 5, H to J). Collectively, these data confirm that BACE1 plays an essential role in LBM cell and tumor growth and provide evidence that targeting BACE1 with the BBB permeable MK-8931 represents a clinically relevant approach to treat BM.

### BACE1 activates the EGFR/MEK/ERK signaling pathway in NSCLC cells

**Fig. 6:**
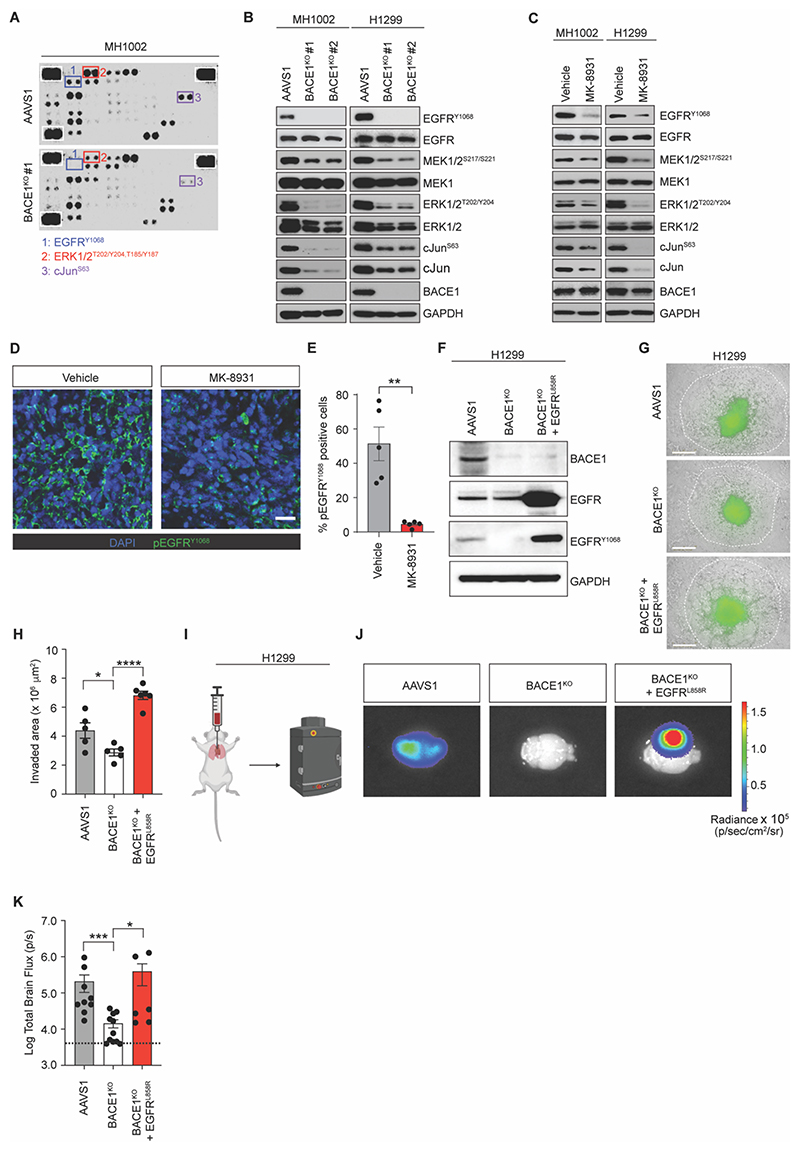
BACE1 activity is required for EGFR activation. (**A**) Human Proteome Profiler^™^ phospho-kinase antibody array in lysates from *BACE1*^KO^ cells. (**B**) Western blot analysis of the indicated proteins in lysates from *BACE1*^KO^ cells (*N*=2). (**C**) Western blot analysis of the indicated proteins in lysates from cells treated with 30 μM MK-8931 for 72 hours (*N*=2). (**D**) Representative micrographs depicting EGFR^Y1068^ staining in MH1002 tumors treated with vehicle or 30 mg/kg MK-8931 (*n*=5 per group, one experiment). Green – EGFR^Y1068^; Blue – DAPI. Scale bar = 30 μm. (**E**) Quantification of proportions of EGFR^Y1068^ positivity in MH1002 tumors in response to vehicle or MK-8931 treatment. (**F**) Western blot analysis of H1299^GFP^ BACE1^KO^ cells restored with EGFR^L858R^ for the indicated proteins (*N*=2). (**G**) Representative micrographs depicting spheroid invasion of BACE1^KO^ H1299^GFP^ with or without EGFR^L858R^ expression after 7 days in Matrigel^™^. White boundaries indicate extent of invasion for the indicated cell lines. Scale bar = 800 μm. (**H**) Quantification of the invaded area of the indicated cell lines in (G) (*n=*5-6, *N*=2). (**I**) Schematic for intracardiac injection of H1299^GFP-Luc^ control, BACE1^KO^ or BACE1^KO + EGFRL858R^ cells. (**J**) Representative *ex vivo* BLI images of brains from the indicated groups (*n*=9-10 per group). (**K**) Quantification of total brain flux from individual mice from the indicated groups. Bars represent mean + SEM. Data in (E) and (H) were analyzed by t test. Data in (K) were analyzed by Mann-Whitney U test. *P<0.05, **P<0.01, ***P<0.001, ****P<0.0001.

BACE1 is the rate-limiting enzyme in the production of Aβ. While critical for the pathogenesis of AD, Aβ has also recently been implicated in playing an instrumental role in the development of melanoma BM(33). To address whether Aβ was involved in our lung cancer system, we measured Aβ secretion into the conditioned media of WT or BACE1-activated CRUK0748-XCL cells (fig. S5A). Interestingly, Aβ concentrations were nearly undetectable in the conditioned media from CRUK0748 cells and decreased when we increased BACE1 expression, suggesting that Aβ is not instrumental in supporting BACE1-dependent LUAD BM in our model (fig. S5A). To uncover the underlying molecular mechanisms by which BACE1 promotes the metastatic potential of LBM cells, we performed a phospho-kinase array screen to identify potential kinases whose activity might be modulated by BACE1. Loss of BACE1 expression resulted in decreased phosphorylation of EGFR, ERK1/2, and cJun (Fig. 6A and fig. S5B). Decreased phosphorylation of EGFR, ERK1/2 and cJun would be consistent with decreased proliferation, self-renewal and migration capacity supporting our earlier findings in the absence of BACE1 (34). We next confirmed the findings of the antibody array by interrogating the associated signaling cascades directly (Fig. 6B). Similar to the antibody array, abrogation of BACE1 expression by CRISPR/Cas9 with two independent sgRNAs resulted in decreased phosphorylation of EGFR, MEK1/2, ERK1/2 and cJun in MH1002 and H1299 cells (Fig. 6B, fig. S5, C and D, and Table S1), indicating the entire MEK/ERK pathway downstream of EGFR was affected by BACE1 expression. Furthermore, we also observed consistent decreases in activation of EGFR, MEK1/2, ERK1/2 and cJun following treatment with MK-8931 in both MH1002 and H1299 cells (Fig. 6C, and fig. S5, E and F). We next interrogated MH1002 tumors that had been treated with MK-8931 for activation of EGFR. While vehicle treated tumors contained considerable EGFR activation marked by tyrosine 1068 phosphorylation, MK-8931 treated tumors contained greatly reduced EGFR activation (Fig. 6, D and E). Together, these data confirm that BACE1 activity is important for maintenance of EGFR/MEK/ERK signaling.

We next sought to confirm this axis was responsible for the brain metastatic phenotype promoted by BACE1 expression. To do this we expressed a constitutively active EGFR (EGFR^L858R^) in BACE1^KO^ H1299^GFP-Luc^ primary LUAD cells (Fig. 6F). The presence of EGFR^L858R^ is one of the most prevalent activating point mutations in EGFR present in patients with EGFR mutant LUAD (35). After confirmation of transgene expression, we evaluated whether constitutive activation of EGFR downstream of BACE1 loss would impact the ability of the cells to invade (Fig. 6G). Indeed, expression of EGFR^L858R^ was able to restore the invasive capacity of H1299^GFP-Luc^ BACE1^KO^ cells (Fig. 6, G and H). We next assessed whether this restored invasive capacity would translate to restoration of a BM phenotype *in vivo*. To do this, we implanted H1299^GFP-Luc^ BACE1^KO^ or BACE1^KO^:EGFR^L858R^ cells directly into the left ventricle of NSG mice (Fig. 6I). At endpoint, bioluminescent imaging of the brains confirmed that BACE1^KO^ cells were unable to efficiently seed BM (Fig. 6, J and K). However, reconstitution of active EGFR in BACE1^KO^ cells (BACE1^KO^:EGFR^L858R^) was able to restore the brain metastatic phenotype of BACE1-deficient cells, demonstrating that restoring EGFR signalling can compensate for the defects in brain metastatic capacity associated with BACE1 loss and the concomitant blunting of the pathway downstream of EGFR. (Fig. 6, J and K).

### EGFR is a novel substrate of BACE1

**Fig. 7:**
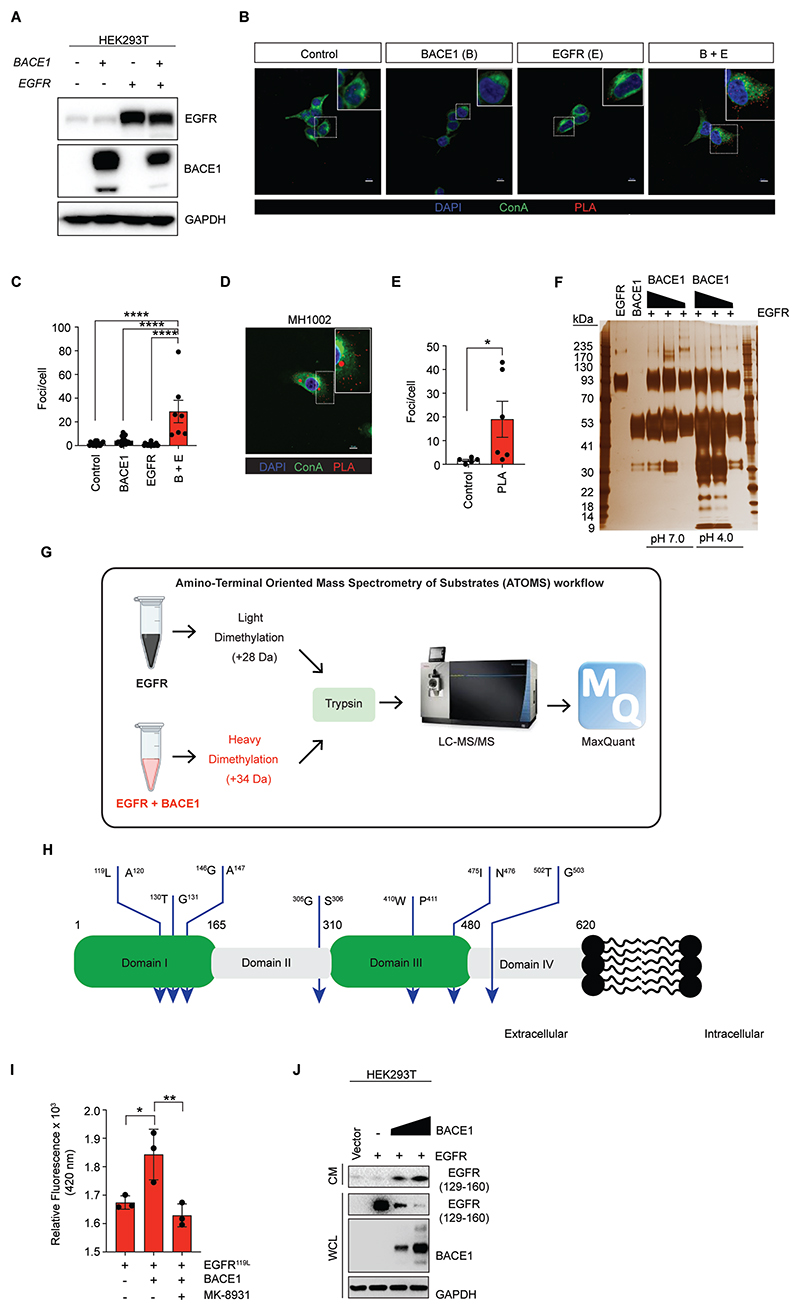
EGFR is a novel substrate of BACE1. (**A**) Western blot analysis of the indicated lysates for BACE1, EGFR (1150-C) and GAPDH following 72 hour expression of the indicated plasmids in HEK293T cells (*N*=2). (**B**) Micrographs depicting proximity-dependent amplification of signal following staining in HEK293T cells expressing *EGFR* and *BACE1* for 72 hours for EGFR and BACE1 (*N*=2). Scale bar = 10 μm. (**C**) Quantification of PLA foci per cell from images in (B). (**D**) Micrographs depicting BACE1 and EGFR PLA signal in MH1002 cells (*N*=2). Scale bar = 10 μm. (**E**) Quantification of PLA foci per cell from images in (D). (**F**) Silver stain analysis following co-incubation of rBACE1 and rEGFR overnight at 37 ºC. 0.25 µg of rEGFR was incubated with 0.5, 2 or 4 µg of rBACE1 in 0.1 M sodium acetate buffer pH 4.0 or 7.0 (*N*=2). (**G**) Schematic of the ATOMS workflow. (**H**) Domain schematic of the ectodomain of EGFR (domains I-IV) highlighting ATOMS identified cleavage sites (blue arrows) following 24 hour co-incubation of rBACE1 and rEGFR (ectodomain) at 37 °C. (**I**) BACE1 FRET activity assay measuring cleavage of EGFR peptide encompassing the ^119^L↓A^120^ cleavage site following co-incubation in the presence or absence of MK-8931 (1 μM) for 24 hours at 37 °C (*N*=2). (**J**) Western blot analysis of the indicated proteins in conditioned media (CM) or whole cell lysates (WCL) from cells transiently expressing the indicated genes for 72 hours (*N*=2). EGFR (129-160) recognizes an epitope near the N-terminus of EGFR between amino acids 129 and 160. Bars in (C) and (E) indicate mean + SEM. Bars in (I) indicate mean + SD. Data in (C) and (I) were analyzed by one-way ANOVA followed by Tukey’s multiple comparisons test. Data in (E) were analyzed by Mann-Whitney U test. *P<0.05, **P<0.01, ****P<0.0001.

Since BACE1 activity in our models has been important for promoting the spread of LUAD to the brain and for influencing cellular biochemical signaling starting from EGFR at the plasma membrane, we next investigated whether the protease activity of BACE1 might have a role in cleaving EGFR. To test this hypothesis, we investigated whether BACE1 and EGFR interacted *in situ* to support a mechanism whereby BACE1 cleaves EGFR directly. We looked into this more closely in HEK293T cells, where neither BACE1 nor EGFR are highly expressed under endogenous conditions (Fig. 7A). We transfected cells with plasmids expressing full length BACE1 and/or EGFR which led to robust expression of both proteins (Fig. 7A). We then performed proximity ligation assays (PLA) to assess whether the two proteins were close enough to physically interact in cells (36). Robust PLA signal was detected in cells expressing both EGFR and BACE1, but not in cells expressing each gene individually (Fig. 7, B and C). To rule out that this was due to over-expression of both BACE1 and EGFR, we performed PLA assays in MH1002 cells that endogenously express BACE1 and EGFR (Fig. 7D). Here again, robust PLA signal was observed, suggesting that BACE1 is in close enough proximity to interact with and mediate direct cleavage of EGFR in cells (Fig. 7, D and E).

To address the possibility that BACE1 could cleave EGFR directly, we co-incubated the recombinant catalytic domain of BACE1 (rBACE1) with the recombinant ectodomain of EGFR (rEGFR) while varying their molar ratios (Fig. 7F). Increasing amounts of rBACE1 led to increasing cleavage of rEGFR when incubated in buffer where BACE1 activity is maximal (pH 4.0). In contrast, when we combined the two proteins together in buffer where rBACE1 is inactive (pH 7.0), with the same molar ratios, we did not detect any cleavage of rEGFR supporting our observations that the cleavage of rEGFR was dependent upon BACE1 activity. To identify the cleavage sites of BACE1 within EGFR we utilized a highly sensitive mass spectrometry technique that would allow for the detection of neo-N-termini (protease cleavage sites) known as amino-terminal oriented mass spectrometry of substrates (ATOMS) (37). A mixture of rEGFR was dimethylated with light formaldehyde (CH_2_O, +28 Da) and a mixture of rEGFR co-incubated with rBACE1 was dimethylated with deuterated/heavy formaldehyde (CD_2_O; +34 Da). Both mixtures were combined and then subjected to LC-MS/MS followed by MaxQuant analysis (Fig. 7G). After a 24 hour co-incubation of rBACE1 and rEGFR, 9 cleavage sites were identified: ^4^S↓G^5^, ^53^R↓M^54^, ^119^L↓A^120^, ^130^T↓G^131^, ^146^G↓A^147^, ^305^G↓S^306^, ^410^W↓P^411^, ^475^I↓N^476^, ^502^T↓G^503^ distributed throughout domains I-IV (Fig. 7H and Table S5). Since a prominent band was detected around ∼10 kDa following co-incubation of rBACE1 and rEGFR, we focused on cleavage sites ^119^L↓A^120^, ^130^T↓G^131^, ^146^G↓A^147^. To validate the cleavage site directly, we measured BACE1 activity towards a peptide encompassing these cleavage sites labelled with FRET donor:acceptor pairs methyl coumarin and dinitrophenol (Fig. 7I and fig. S6A). Co-incubation of rBACE1 with the fluorogenic peptide containing the ^119^L↓A^120^ cleavage site, but not the peptide alone, produced fluorescent signal, which was dependent upon BACE1 activity as MK-8931 treatment blocked the production of a detectable fluorescent signal (Fig. 7I). Moreover, no BACE1 activity could be detected towards ^130^T↓G^131^, ^146^G↓A^147^ sites during the reaction suggesting the ^119^L↓A^120^ cleavage site was the predominant cleavage site in that region of the protein (fig. S6A). Together, these data confirm EGFR to be a novel substrate of BACE1.

Next, we addressed whether BACE1 could cleave EGFR in cells. To do this, we once again transiently expressed BACE1 and EGFR in HEK293T cells (Fig. 7J). Probing for EGFR with an antibody specific to an N-terminal epitope consisting of amino acids 129-160 revealed EGFR expression in cell lysates decreased as BACE1 expression increased. Next, we probed conditioned medium from cells in which these lysates were collected for the N-terminal fragment of EGFR (129-160) and observed a concomitant increase in the presence of this N-terminal peptide in response to increasing amounts of BACE1 expression in these cells (Fig. 7J), strongly suggesting that BACE1 was mediating the cleavage of EGFR in cells. Based on our findings, BACE1 inhibition would not be predicted to have much effect on the growth of EGFR mutant LUAD lines. To test this directly, we treated EGFR mutant LUAD lines PC9 and H1975 with MK-8931 (fig. S6, B to D). Indeed, MK-8931 treatment did not have much of an effect of the growth of these lines. Furthermore, since Osimertinib is administered in a front-line setting to patients with EGFR mutant LUAD (5), we tested whether there was any potential interaction between the two drugs in these models (fig. S6, E and F). In agreement with our MK-8931 studies, and data up to this point, there was a lack of any synergy between the two drugs. However, importantly there was also absence of any antagonistic effects of the two drugs suggesting that they may be co-administered without any impact on the efficacy of EGFR inhibition.

In summary, our findings support a model whereby BACE1 promotes LUAD brain metastasis *via* its novel substrate EGFR.

## Discussion

There are very few effective treatment options available for patients with BM. In this study, we developed an *in vivo* CRISPRa screen in an orthotopic patient-derived LUAD model to identify putative drivers of BM which could potentially serve as future biomarkers and/or therapeutic targets for this deadly disease. We identified BACE1 as a critical enzyme involved in enhancing the invasive, proliferative and initiation capacity of primary and brain metastatic LUAD cells by activating the EGFR/MEK/ERK cellular signaling axis. Our study thus identifies a new therapeutic target to be explored for BM.

Verubecestat (MK-8931) is the first BACE1 inhibitor to advance to phase 3 clinical trial (38) and is generally safe and well-tolerated in healthy adults (39) and AD patients (29, 31). Although targeting BACE1 by Verubecestat is not effective for treating AD patients (29), our studies and the fact that this drug is blood-brain barrier penetrant, strongly indicate that it can be repurposed for treatment of BM, highlighting the potential of BACE1 as a novel target for treating lung cancer. In support of our findings, Aβ secretion from melanoma cells was recently shown to facilitate the growth of melanoma cells in the brain which could be reduced through the use of a BACE inhibitor (33). While our study does not support a role of amyloid precursor protein (APP) or Aβ in BACE1-mediated BM in our models, the importance of BACE1 in modulating EGFR signaling is clear, and future work to evaluate the utility of Aβ as a biomarker for lung cancer BM as a surrogate for BACE1 expression/activity is warranted.

EGFR plays a fundamental role in cancer biology and especially NSCLC biology (40). Here we identify EGFR is a novel substrate of BACE1. Cleavage of EGFR between L^119^ and A^120^ is predicted to disrupt the four interactions EGF makes with the receptor in domain I at L^14^, Y^45^, L^69^ and L^98^ (41). While this cleavage might induce conformational changes to influence ligand binding, receptor dimerization and activation, future studies are needed to fully appreciate the biophysical and biochemical changes to EGFR following cleavage by BACE1. The implications of this finding are far reaching, as therapeutic targeting of EGFR in NSCLC has revolutionized patient care, greatly extending survival times over chemotherapy, although drug resistance remains a challenge (5). Here, we demonstrate that in instances where EGFR is wild-type, which accounts for more than half of all patients with LUAD (40), BACE1 is needed for activation of EGFR and downstream signaling. This suggests that a patient population otherwise not being considered for EGFR-targeting tyrosine kinase inhibitors in a front-line setting due to lack of mutations detected by sequencing, may benefit from such a therapy if BACE1 is present in their tumor. Based on our findings, therapeutic targeting of BACE1 may not be predicted to benefit patients with activating mutations in EGFR; however, our data in EGFR mutant LUAD models indicates MK-8931 and osimertinib are not antagonistic suggesting that the two may be co-administered without any deleterious effects of one drug on the other’s efficacy. Nevertheless, our data are supportive of targeting BACE1 in KRAS driven lung cancers which also have a strong predilection to end up in the brain (42). Evidence exists in other cancers demonstrating EGFR activity is needed for full activation of MEK and ERK downstream of mutant KRAS and this may be the first example of that in NSCLC (43), reinforcing a need to target BACE1 in this setting.

A limitation to our study is that our models are patient-derived and engrafted in immunocompromised mice omitting the contribution of the immune system to the development of BM. However, since our screen was conducted with these models and identified the cell autonomous role of BACE1 in the development of lung cancer brain metastasis, these findings are likely to be conserved in the presence of immune cell populations. Nevertheless, addressing the contribution of the immune system to our findings will be the subject of future investigations.

In conclusion, our *in vivo* CRISPR activation screen identified BACE1 as a novel, tractable target for LUAD BM. In light of these findings, we suggest repurposing drugs designed to target BACE1 for AD, in particular Verubecestat, in LUAD to suppress the development of BM.

## Materials and Methods

### Study design

This study was designed to identify genes promoting the spread of NSCLC to the brain. Preclinical patient-derived animal models of NSCLC and BM that arose from NSCLC were utilized to investigate the role of BACE1 in BM and for therapeutic intervention studies. Sample sizes were chosen based on effect sizes based on pilot experiments and no statistical method was used to predetermine sample size. The indicated sample size represents biological replicates and each experiment was performed two to three times. Replicates were only excluded if indicated to be true outliers by the Grubb’s test. For *in vivo* studies mice were randomly assigned to treatment group. Investigators were not blinded to treatment group during data collection and analysis.

### Statistics

All bar graphs plot the mean ± SEM or mean ± SD as indicated. Significant differences were determined between two groups using the two-way Student’s t-test and Mann-Whitney U test for non-parametric data or among multiple groups using one-way ANOVA with Sidak’s or Tukey’s multiple comparisons tests *post-hoc* or two-way ANOVA and statistical significance was set at *p* < 0.05. Survival analysis was performed using the Log-rank test. All analyses were performed with GraphPad Prism 9 software (https://www.graphpad.com/). Multivariate analyses were run using a cox proportional hazards model in R using age, sex, stage and smoking status as covariates (51). Experimental details such as number of animals or cells and experimental replication were provided in the figure legends. Data inclusion/exclusion criteria was not applied in this study.

## Supplementary Material

movie s1

movie s2

table s1

## Data Availability

All data associated with this study are in the paper or the supplementary materials. All sequencing and proteomic data have been deposited into their appropriate databases. All FASTQ files from the CRISPR screen have been deposited in the Gene Expression Omnibus (GEO) under accession # GSE237446. All proteomic data has been deposited in the PRIDE database under accession # PXD060790. All requests for reagents will be fulfilled by SKS or SB following completion of material transfer agreements with McMaster University (SKS) or the Cleveland Clinic (SB).

## One sentence summary

*in vivo* CRISPR activation screen identifies beta secretase 1 (BACE1) promotes the spread of lung cancers to the brain *via* EGFR signaling.

## References

[1] Aizer AA, Lamba N, Ahluwalia MS, Aldape K, Boire A, Brastianos PK, Brown PD, Camidge DR, Chiang VL, Davies MA, Hu LS (2022). Brain metastases: A Society for Neuro-Oncology (SNO) consensus review on current management and future directions. Neuro Oncol.

[2] Achrol AS, Rennert RC, Anders C, Soffietti R, Ahluwalia MS, Nayak L, Peters S, Arvold ND, Harsh GR, Steeg PS, Chang SD (2019). Brain metastases. Nat Rev Dis Primers.

[3] Cagney DN, Martin AM, Catalano PJ, Redig AJ, Lin NU, Lee EQ, Wen PY, Dunn IF, Bi WL, Weiss SE, Haas-Kogan DA (2017). Incidence and prognosis of patients with brain metastases at diagnosis of systemic malignancy: a population-based study. Neuro Oncol.

[4] Janne PA, Riely GJ, Gadgeel SM, Heist RS, Ou SI, Pacheco JM, Johnson ML, Sabari JK, Leventakos K, Yau E, Bazhenova L (2022). Adagrasib in Non-Small-Cell Lung Cancer Harboring a KRAS(G12C) Mutation. N Engl J Med.

[5] Soria JC, Ohe Y, Vansteenkiste J, Reungwetwattana T, Chewaskulyong B, Lee KH, Dechaphunkul A, Imamura F, Nogami N, Kurata T, Okamoto I (2018). Osimertinib in Untreated EGFR-Mutated Advanced Non-Small-Cell Lung Cancer. N Engl J Med.

[6] Brastianos PK, Carter SL, Santagata S, Cahill DP, Taylor-Weiner A, Jones RT, Van Allen EM, Lawrence MS, Horowitz PM, Cibulskis K, Ligon KL (2015). Genomic Characterization of Brain Metastases Reveals Branched Evolution and Potential Therapeutic Targets. Cancer Discov.

[7] Shih DJH, Nayyar N, Bihun I, Dagogo-Jack I, Gill CM, Aquilanti E, Bertalan M, Kaplan A, D’Andrea MR, Chukwueke U, Ippen FM (2020). Genomic characterization of human brain metastases identifies drivers of metastatic lung adenocarcinoma. Nat Genet.

[8] Singh M, Venugopal C, Tokar T, Brown KR, McFarlane N, Bakhshinyan D, Vijayakumar T, Manoranjan B, Mahendram S, Vora P, Qazi M (2017). RNAi screen identifies essential regulators of human brain metastasis-initiating cells. Acta Neuropathol.

[9] Nguyen DX, Chiang AC, Zhang XH, Kim JY, Kris MG, Ladanyi M, Gerald WL, Massague J (2009). WNT/TCF signaling through LEF1 and HOXB9 mediates lung adenocarcinoma metastasis. Cell.

[10] Singh M, Garg N, Venugopal C, Hallett R, Tokar T, McFarlane N, Mahendram S, Bakhshinyan D, Manoranjan B, Vora P, Qazi M (2015). STAT3 pathway regulates lung-derived brain metastasis initiating cell capacity through miR-21 activation. Oncotarget.

[11] Valiente M, Obenauf AC, Jin X, Chen Q, Zhang XH, Lee DJ, Chaft JE, Kris MG, Huse JT, Brogi E, Massague J (2014). Serpins promote cancer cell survival and vascular co-option in brain metastasis. Cell.

[12] Bassey-Archibong BI, Rajendra Chokshi C, Aghaei N, Kieliszek AM, Tatari N, McKenna D, Singh M, Kalpana Subapanditha A, Parmar A, Mobilio D, Savage N (2023). An HLA-G/SPAG9/STAT3 axis promotes brain metastases. Proc Natl Acad Sci U S A.

[13] Hynds RE, Huebner A, Pearce DR, Hill MS, Akarca AU, Moore DA, Ward S, Gowers KHC, Karasaki T, Al Bakir M, Wilson GA (2024). Representation of genomic intratumor heterogeneity in multi-region non-small cell lung cancer patient-derived xenograft models. Nat Commun.

[14] Qi LS, Larson MH, Gilbert LA, Doudna JA, Weissman JS, Arkin AP, Lim WA (2013). Repurposing CRISPR as an RNA-guided platform for sequence-specific control of gene expression. Cell.

[15] Mali P, Aach J, Stranges PB, Esvelt KM, Moosburner M, Kosuri S, Yang L, Church GM (2013). CAS9 transcriptional activators for target specificity screening and paired nickases for cooperative genome engineering. Nat Biotechnol.

[16] Sanson KR, Hanna RE, Hegde M, Donovan KF, Strand C, Sullender ME, Vaimberg EW, Goodale A, Root DE, Piccioni F, Doench JG (2018). Optimized libraries for CRISPR-Cas9 genetic screens with multiple modalities. Nat Commun.

[17] Kieliszek AM, Mobilio D, Bassey-Archibong BI, Johnson JW, Piotrowski ML, de Araujo ED, Sedighi A, Aghaei N, Escudero L, Ang P, Gwynne WD (2024). De novo GTP synthesis is a metabolic vulnerability for the interception of brain metastases. Cell Rep Med.

[18] Tsherniak A, Vazquez F, Montgomery PG, Weir BA, Kryukov G, Cowley GS, Gill S, Harrington WF, Pantel S, Krill-Burger JM, Meyers RM (2017). Defining a Cancer Dependency Map. Cell.

[19] Wei S, Liu W, Xu M, Qin H, Liu C, Zhang R, Zhou S, Li E, Liu Z, Wang Q (2022). Cathepsin F and Fibulin-1 as novel diagnostic biomarkers for brain metastasis of non-small cell lung cancer. Br J Cancer.

[20] Sevenich L, Bowman RL, Mason SD, Quail DF, Rapaport F, Elie BT, Brogi E, Brastianos PK, Hahn WC, Holsinger LJ, Massague J (2014). Analysis of tumour- and stroma-supplied proteolytic networks reveals a brain-metastasis-promoting role for cathepsin S. Nat Cell Biol.

[21] Ferraro GB, Ali A, Luengo A, Kodack DP, Deik A, Abbott KL, Bezwada D, Blanc L, Prideaux B, Jin X, Posada JM (2021). Fatty Acid Synthesis Is Required for Breast Cancer Brain Metastasis. Nat Cancer.

[22] Jin X, Demere Z, Nair K, Ali A, Ferraro GB, Natoli T, Deik A, Petronio L, Tang AA, Zhu C, Wang L (2020). A metastasis map of human cancer cell lines. Nature.

[23] Nolte SM, Venugopal C, McFarlane N, Morozova O, Hallett RM, O’Farrell E, Manoranjan B, Murty NK, Klurfan P, Kachur E, Provias JP (2013). A cancer stem cell model for studying brain metastases from primary lung cancer. J Natl Cancer Inst.

[24] Singh M, Venugopal C, Tokar T, McFarlane N, Subapanditha MK, Qazi M, Bakhshinyan D, Vora P, Murty NK, Jurisica I, Singh SK (2018). Therapeutic Targeting of the Premetastatic Stage in Human Lung-to-Brain Metastasis. Cancer Res.

[25] Singh SK, Hawkins C, Clarke ID, Squire JA, Bayani J, Hide T, Henkelman RM, Cusimano MD, Dirks PB (2004). Identification of human brain tumour initiating cells. Nature.

[26] Coleman KE, Bekes M, Chapman JR, Crist SB, Jones MJ, Ueberheide BM, Huang TT (2017). SENP8 limits aberrant neddylation of NEDD8 pathway components to promote cullin-RING ubiquitin ligase function. Elife.

[27] Hellyer JA, Stehr H, Das M, Padda SK, Ramchandran K, Neal JW, Diehn M, Wakelee HA (2019). Impact of KEAP1/NFE2L2/CUL3 mutations on duration of response to EGFR tyrosine kinase inhibitors in EGFR mutated non-small cell lung cancer. Lung Cancer.

[28] Zhai K, Huang Z, Huang Q, Tao W, Fang X, Zhang A, Li X, Stark GR, Hamilton TA, Bao S (2021). Pharmacological inhibition of BACE1 suppresses glioblastoma growth by stimulating macrophage phagocytosis of tumor cells. Nat Cancer.

[29] Naidu A, Silverglate B, Silverglate M, Grossberg GT (2025). Safety concerns associated with BACE1 inhibitors - past, present, and future. Expert Opin Drug Saf.

[30] Zhang Q, Abdo R, Iosef C, Kaneko T, Cecchini M, Han VK, Li SS (2022). The spatial transcriptomic landscape of non-small cell lung cancer brain metastasis. Nat Commun.

[31] Kennedy ME, Stamford AW, Chen X, Cox K, Cumming JN, Dockendorf MF, Egan M, Ereshefsky L, Hodgson RA, Hyde LA, Jhee S (2016). The BACE1 inhibitor verubecestat (MK-8931) reduces CNS beta-amyloid in animal models and in Alzheimer’s disease patients. Sci Transl Med.

[32] Clarke MF, Dick JE, Dirks PB, Eaves CJ, Jamieson CH, Jones DL, Visvader J, Weissman IL, Wahl GM (2006). Cancer stem cells--perspectives on current status and future directions: AACR Workshop on cancer stem cells. Cancer Res.

[33] Kleffman K, Levinson G, Rose IVL, Blumenberg LM, Shadaloey SAA, Dhabaria A, Wong E, Galan-Echevarria F, Karz A, Argibay D, Von Itter R (2022). Melanoma-Secreted Amyloid Beta Suppresses Neuroinflammation and Promotes Brain Metastasis. Cancer Discov.

[34] Guo YJ, Pan WW, Liu SB, Shen ZF, Xu Y, Hu LL (2020). ERK/MAPK signalling pathway and tumorigenesis. Exp Ther Med.

[35] Sharma SV, Bell DW, Settleman J, Haber DA (2007). Epidermal growth factor receptor mutations in lung cancer. Nat Rev Cancer.

[36] Soderberg O, Gullberg M, Jarvius M, Ridderstrale K, Leuchowius KJ, Jarvius J, Wester K, Hydbring P, Bahram F, Larsson LG, Landegren U (2006). Direct observation of individual endogenous protein complexes in situ by proximity ligation. Nat Methods.

[37] Doucet A, Overall CM (2011). Amino-Terminal Oriented Mass Spectrometry of Substrates (ATOMS) N-terminal sequencing of proteins and proteolytic cleavage sites by quantitative mass spectrometry. Methods Enzymol.

[38] Yan R, Vassar R (2014). Targeting the beta secretase BACE1 for Alzheimer’s disease therapy. Lancet Neurol.

[39] Forman M, Palcza J, Tseng J, Stone JA, Walker B, Swearingen D, Troyer MD, Dockendorf MF (2019). Safety, Tolerability, and Pharmacokinetics of the beta-Site Amyloid Precursor Protein-Cleaving Enzyme 1 Inhibitor Verubecestat (MK-8931) in Healthy Elderly Male and Female Subjects. Clin Transl Sci.

[40] Skoulidis F, Heymach JV (2019). Co-occurring genomic alterations in non-small-cell lung cancer biology and therapy. Nat Rev Cancer.

[41] Ogiso H, Ishitani R, Nureki O, Fukai S, Yamanaka M, Kim JH, Saito K, Sakamoto A, Inoue M, Shirouzu M, Yokoyama S (2002). Crystal structure of the complex of human epidermal growth factor and receptor extracellular domains. Cell.

[42] Lamberti G, Aizer A, Ricciuti B, Alessi JV, Pecci F, Tseng SC, Sholl LM, Nishino M, Awad MM (2023). Incidence of Brain Metastases and Preliminary Evidence of Intracranial Activity With Sotorasib in Patients With KRAS(G12C)-Mutant Non-Small-Cell Lung Cancer. JCO Precis Oncol.

[43] Ardito CM, Gruner BM, Takeuchi KK, Lubeseder-Martellato C, Teichmann N, Mazur PK, Delgiorno KE, Carpenter ES, Halbrook CJ, Hall JC, Pal D (2012). EGF receptor is required for KRAS-induced pancreatic tumorigenesis. Cancer Cell.

[44] Galaxy C (2024). The Galaxy platform for accessible, reproducible, and collaborative data analyses: 2024 update. Nucleic Acids Res.

[45] Ianevski A, Giri AK, Aittokallio T (2022). SynergyFinder 3.0: an interactive analysis and consensus interpretation of multi-drug synergies across multiple samples. Nucleic Acids Res.

[46] Law AMK, Yin JXM, Castillo L, Young AIJ, Piggin C, Rogers S, Caldon CE, Burgess A, Millar EKA, O’Toole SA, Gallego-Ortega D (2017). Andy’s Algorithms: new automated digital image analysis pipelines for FIJI. Sci Rep.

[47] Li B, Dewey CN (2011). RSEM: accurate transcript quantification from RNA-Seq data with or without a reference genome. BMC Bioinformatics.

[48] Martinez-Ruiz C, Black JRM, Puttick C, Hill MS, Demeulemeester J, Larose Cadieux E, Thol K, Jones TP, Veeriah S, Naceur-Lombardelli C, Toncheva A (2023). Genomic-transcriptomic evolution in lung cancer and metastasis. Nature.

[49] Frankell AM, Dietzen M, Al Bakir M, Lim EL, Karasaki T, Ward S, Veeriah S, Colliver E, Huebner A, Bunkum A, Hill MS (2023). The evolution of lung cancer and impact of subclonal selection in TRACERx. Nature.

[50] Forbes SA, Beare D, Gunasekaran P, Leung K, Bindal N, Boutselakis H, Ding M, Bamford S, Cole C, Ward S, Kok CY (2015). COSMIC: exploring the world’s knowledge of somatic mutations in human cancer. Nucleic Acids Res.

[51] Wang X, Bai H, Zhang J, Wang Z, Duan J, Cai H, Cao Z, Lin Q, Ding X, Sun Y, Zhang W (2024). Genetic Intratumor Heterogeneity Remodels the Immune Microenvironment and Induces Immune Evasion in Brain Metastasis of Lung Cancer. J Thorac Oncol.

