# Supplementary material for: Genome-wide in vivo CRISPR activation screen identifies BACE1 as a therapeutic vulnerability of lung cancer brain metastasis
Source: Sci Transl Med. Author manuscript; Available in PMC 2025 Aug 7. (PMC7617989; doi:10.1126/scitranslmed.adu2459)
Supplement: table s1 [file EMS207657-supplement-table_s1.pdf]

Science Translational Medicine
AAAS

Supplementary Materials for

A genome-wide in vivo CRISPR activation screen identifies BACE1 as a therapeutic vulnerability of lung cancer brain metastasis

Shawn C. Chafe *et al.*

Corresponding author: Sheila K. Singh, ssingh@mcmaster.ca; Shideng Bao, baos@ccf.org

Sci. Transl. Med. **17**, eadu2459 (2025)
DOI: 10.1126/scitranslmed.adu2459

The PDF file includes:

Materials and Methods
Figs. S1 to S6
Tables S1 and S2
Legends for movies S1 and S2
Legends for data files S1 to S6
References (*45–51*)

Other Supplementary Material for this manuscript includes the following:

Movies S1 and S2
Data files S1 to S6
MDAR Reproducibility Checklist

Materials and Methods

Cell lines

The human lung cancer cell lines, A549 (RRID: CVCL_A549) and H1573 (RRID: CVCL_1478), were obtained from the American Type Culture Collection (ATCC). The H1299 (RRID: CVCL_0060) human lung cancer cell lines were kindly gifted by Dr. Andrei Ivanov from the Lerner Research Institute at Cleveland Clinic. The PC9 (RRID: CVCL_B260) and H1975 (RRID: CVCL_1511) cells were generous gifts from Dr. Don Nguyen from the Yale School of Medicine. The MH1002, MH1012, BT530, BT478, and MBT456 patient-derived cell lines were generated in our own laboratories from metastatic brain specimens of lung adenocarcinoma (LUAD) origin. The primary LUAD lines, CRUK0748-XCL and CRUK0733-XCL, were derived from subcutaneous patient-derived xenograft models established within the TRACERx study following serial implantation in NOD.Cg-Prkdc^{SCID} IL2rg^{tm1Wjl}/SzJ (NSG) mice (*13*). All lung cancer cell lines were maintained in neurobasal medium (Invitrogen, 12349015) supplemented with B-27 (Invitrogen, 12587010), 2 mM glutamine (Thermo Fisher, 35050061), non-essential amino acids (NEAA) (Thermo Fisher, 11140050), 1 mM sodium pyruvate (Thermo Fisher, 11360070), 20 ng/mL epidermal growth factor (EGF, Goldbio, 1150-04-100), and 20 ng/mL basic fibroblast growth factor (bFGF, R&D Systems, 4114-TC-01M). The 293FT cells (Clontech; 632180, RRID: CVCL_6911) were maintained in Dulbecco's modified Eagle medium (DMEM) supplemented with 10% fetal bovine serum (FBS). All cells used in this study were consistently confirmed to be free from mycoplasma by using a MycoFluor Mycoplasma Detection Kit (Thermo Fisher, M7006). Unless otherwise

indicated, media was supplemented with antibiotic-antimycotic solution (Thermo Fisher, 15240062) to prevent contamination.

Chemicals and reagents

MK-8931 was purchased from Selleckchem (S8173) or Medkoo (331024). AZD3293 (S8193) and AZD3839 (S7731) were purchased from Selleckchem. PF-06751979 (555239) was purchased from Medkoo. Osimertinib was purchased from Selleckchem (S7297). D-Luciferin was purchased from GoldBio (LUCK-10G) or Perkin Elmer (122799). 32% Paraformaldehyde (PFA, 15714) was from Electron Microscopy Sciences and diluted to 4% with phosphate-buffered saline (PBS) before use. Protease (04693159001) and phosphatase inhibitor (04906837001) tablets were from Roche. All other chemicals and reagents were purchased from Sigma-Aldrich.

Human surgical specimens

The human surgical specimens of human lung cancer brain metastases were collected from the Brain Tumor and Neuro-Oncology Center at Cleveland Clinic according to a protocol approved by the Cleveland Clinic Institutional Review Broad as well as from the Hamilton General Hospital and St. Joseph's Healthcare Hamilton according to a protocol approved by the Hamilton Integrated Research Ethics Board (HiREB #4917). The surgical specimens were used for isolation of lung cancer cells, immunohistochemical analyses, or immunofluorescent analyses. The tissue microarray of primary lung cancer (LC1923) was from US Biomax Inc. The tissue microarray of 21 matched primary lung cancer lung

tumors and their corresponding metastatic brain tumors was previously described (*30*). The fidelity of all patient samples was confirmed by a pathologist (J.,-Q.,L; J.,-C.,C, Q.Z).

Plasmids

The human *BACE1* sgRNA CRISPR/Cas9 All-in-One Lentivectors (K0166207 and K0166208) and the scrambled sgRNA CRISPR/Cas9 All-in-One Lentivector (K010) were purchased from ABM. The vectors for expressing human *EGFR* ORF (EX-A8661-Lv158) or *BACE1* ORF (EX-U0498-Lv128) were purchased from GeneCopoeia. The dCas9-VP64-BLAST (pXPR_109)(Addgene #61425, RRID: Addgene_61425) and the $EGFR^{L858R}$ (pHAGE-$EGFR^{L858R}$)(Addgene #116276, RRID: Addgene_116276) plasmids were purchased from Addgene. The lentiviral GFP-luciferase plasmid was a kind gift from Fred C. Lam.

Library preparation

Human Calabrese CRISPR activation pooled library set A was a gift from David Root and John Doench (Addgene #92379). This pooled plasmid library was used to produce lentivirus as previously described (*16*). Briefly, twenty 80% confluent 150 mm dishes of HEK293T cells (approximately 12 million cells per plate) were transfected with pCMV-VSVG (4.2 μg) (Addgene #8454, RRID: Addgene_8454), psPAX2 (42 μg) (Addgene #12260, RRID: Addgene_12260), library plasmid pool (33.3 μg) (Addgene #92379, RRID: Addgene_92379), and XtremeGENE 9 (238.5 μL) (Roche), and mixed in a total of 2 mL OptiMEM (Thermo Fisher) and added to each plate of HEK293T cells containing (D10V) DMEM containing 10% FBS, 1x NEAA, 1 mM HEPES and 1 mM sodium butyrate (Sigma

#B5887). Media was harvested and exchanged with fresh media on days 2 to 4. On day 5, viral particles were pelleted by ultracentrifugation at 20,000 rpm for two hours at 4°C, and concentrated virus was stored at 4°C overnight, followed by long-term storage at -80°C.

Generation of CRISPR activation cell lines

CRUK0733-XCL and CRUK0748-XCL cell lines were transduced with lentivirus encoding dCas9-VP64 (D). Transduced cells were selected with blasticidin. Once expanded, CRUK0733-XCL-D and CRUK0748-XCL-D cells were transduced with lentivirus encoding GFP-luciferase (GL). Transduced cells were selected by flow cytometry and sorted by fluorescence activated cell sorting (FACS). CRUK0733-XCL-GLD and CRUK0748-XCL-GLD cells were used in CRISPR activation studies.

Flow Cytometry

1×10^6 CRUK0748-XCL-GLD cells expressing control or *PTPRC* targeting sgRNA were stained with anti-CD45 allophycocyanin (APC)-Cyanine (Cy) 7 antibody (BioLegend, 368515, clone 2D1, RRID: AB_2566375, 1:50) for 20 minutes at room temperature in staining buffer (PBS, pH 7.4, containing 2 mM EDTA). Cells were washed with staining buffer and stained with 7AAD (BioLegend, 420403, 1:100). Viable cells were analyzed on a CytoFlex (Beckman Coulter) for CD45 expression.

CRISPRa screen

To achieve 500X representation of the Calabrese library A, we deduced that we needed a final cell population of $5x10^7$ cells infected with the library to be injected into the lungs of 30 mice ($2x10^6$ cells per mouse lung). CRUK0748-XCL-GLD cells were transduced with Calabrese Library A lentivirus at a multiplicity of infection (MOI) of approximately 0.3 overnight and then selected with puromycin for 72 hours. Following selection, plates were split and cells allowed to grow for 48 hours in media free of puromycin to recover prior to inoculation of mice. At this point, $3x10^7$ cells were harvested for a T_0 cell pellet (initial screen timepoint) and frozen at -80°C to be sequenced at a later stage, and $6x10^7$ cells were prepared for inoculation of mice. The cell suspension was then prepared to deliver $2x10^6$ cells per mouse in a final volume of 70 μL in PBS containing 10% Matrigel (Corning). In parallel, CRUK0748-XCL-GLD cells from control plates exposed to neither Calabrese A virus nor puromycin were dissociated and prepared for control injections (*n*=4). Library-transduced cells were then injected into 30 NSG mice, representing greater than 500x coverage of the library, through the modified thoracotomy orthotopic intrathoracic injection (see below). Tumor burden was monitored weekly by bioluminescent imaging (BLI) until mice became moribund (day 26 to 29), at which time mice were injected with 150 mg/kg d-luciferin and euthanized, then lungs and brains removed and brains imaged by BLI and then flash frozen until genomic DNA could be extracted.

Genomic DNA extraction and sequencing

Genomic DNA was extracted from lung and brain tissue using the Gentra Puregene Tissue kit from Qiagen (#158689). Isolated DNA was phenol:chloroform extracted and ethanol precipitated to improve DNA quality before proceeding. sgRNAs were amplified using previously described primers (also listed in table S2) and reaction conditions (*16*) using PrimeStar GXL DNA polymerase (TakaraBio, R050A). Twelve brain samples and 11 lung samples were sequenced on a NovaSeq SP flow cell with a paired-end 100 bp kit with 10-15% PhiX DNA spike in performed by The Centre for Applied Genomics, The Hospital for Sick Children, Toronto, Canada. Each sample received 30 to 40 million reads and indexed reads were demultiplexed prior to analysis.

CRISPRa screen data analysis

Reads underwent quality assurance, end-trimming (cutadapt and trim galore) using the Galaxy toolshed (*45*). Reads were then aligned to the Calabrese Library A index file using Bowtie v1.3.1 allowing for single nucleotide mismatches and discarding reads that aligned to more than a single sgRNA. Aligned reads for each sample were then assembled into a read matrix file and analyzed using R. sgRNA read counts were normalized to one million reads per sample and were then averaged across all lungs and brains to determine the mean normalized read count per sgRNA. The mean read counts per sgRNA were then averaged for the top 2 abundant sgRNAs targeting each gene to determine the mean normalized sgRNA read count per gene. This was determined for both lungs and brains. The mean normalized read count per gene was utilized when determining the fold change (FC) in normalized sgRNA read counts per tissue per animal relative to control sgRNAs

and plotted as rug plots. FC = (normalized read count targeting sgRNA/mean normalized read count control sgRNA). The normalized read counts per gene were also expressed as fold change relative to the control sgRNAs in their respective tissue. This was determined for both lungs and brains to determine the relative increase in abundance of each sgRNA in the brains relative to the lungs and plotted as violin plots.

Lentivirus production and *BACE1* knockout cell generation

Lentiviruses were produced in 293FT cells and prepared as previously described (*28*). Briefly, 293FT cells were co-transduced with targeting plasmids and packaging vectors pCMV-VSVG and psPAX2 by using PEI (Serochem, AQ100). Four days after transfection, the supernatants were harvested and virus titers were determined as described previously (*28*). For infection, cells were treated with lentivirus at an MOI of 1. CRISPR/Cas9 was used to generate *BACE1* KO lung cancer cells. Briefly, MH1002 or H1299 cells were infected by lentiviruses expressing human *BACE1* sgRNA CRISPR/Cas9 All-in-One Lentivectors or the scrambled sgRNA CRISPR/Cas9 All-in-One Lentivector (K010) for 12 hours. Two days after infection, the cells were treated with 2 μg/mL puromycin (Fisher Scientific, BP2956100) for 7 days. After treatment, single-cell clones were cultured. Immunoblot analysis as described below confirmed successful knockout of *BACE1*.

Patient derived xenograft (PDX) establishment and drug treatment in vivo

All animal experiments were performed in accordance with protocols approved by the IACUC of the Lerner Research Institute at the Cleveland Clinic (AUP# 2559) and the

AREB (AUP# 22-12-38) of McMaster University. Six- to 8-week old NSG mice were used for establishing PDXs derived from human lung cancer cells for the in vivo studies. Mice were maintained in a 12-hour light/12-hour dark cycle, and provided with sterilized water and food ad libitum at the Biological Resource Unit of the Cleveland Clinic Lerner Research Institute or the Central Animal Facility (CAF) of McMaster University.

To establish xenografts for in vivo studies, transplantation of wild type (WT) or *BACE1* KO MH1002 cells into the brains of NSG mice was performed as described previously (*17*). In vivo bioluminescent imaging was performed twice per week to monitor tumor growth, using the Spectrum CT Imaging System (Perkin Elmer), before and after treatment. For drug treatment, a stock solution of MK-8931 at 100 mg/mL in dimethyl sulfoxide (DMSO) was diluted in 0.5% (w/v) methylcellulose (Sigma-Aldrich, M0512) to 6 mg/mL (*31*). Mice bearing xenografts from WT MH1002 cells or CRISPR activated-*BACE1* CRUK0748-XCL-GLD cells were treated with MK-8931 (30 mg/kg) or the control (DMSO) once daily by oral gavage for three weeks or until humane endpoint was reached. To collect mouse brains bearing tumors, cardiac perfusion with PBS and 4% PFA was performed. The brains were fixed and sectioned for further immunofluorescent, histochemical and histological analyses.

For the thoracotomy model, mice were anesthetized with isoflurane and provided with pre-operative buprenorphine (0.5 mg/kg), carprofen (5 mg/kg) and saline. Mice were then immobilized with the right forelimb immobilized above the head to expose the right chest. Fur was removed over the surgical site with scissors and the surgical site was cleaned

with 7% followed by 10% iodine scrub. A 1 cm incision was made in the skin over the rib cage to visualize the lungs. A superficial injection of the cell suspension ($5x10^5$ cells) in 10% Matrigel was made between the ribs directly into the lung. The wound was sutured and glued closed with tissue glue and the mice were allowed to recover on heatpads in fresh cages. Metastatic growth was determined by ex vivo BLI of brains, livers, and leg bones and visualized with petal plots using the ggplot2 function in R. Petal height indicates average total bioluminescent flux (0 to 10^8) for the group for that organ. Petal width (0 to 1) depicts the metastatic penetrance for the group.

For the intracardiac model, mice were anesthetized with isoflurane. Mice were then immobilized in the supine position with forelimbs immobilized overhead. The chest was sterilized with 70% ethanol and an injection was made 3 mm to the left of midline in line with the left axilla. $2x10^5$ cells in sterile saline were slowly administered with a 0.5 cc insulin syringe (29G x ½") in 50 μL. Mice were allowed to recover on heatpads in fresh cages.

Immunofluorescence and immunohistochemistry

Immunofluorescent staining of tumor tissues or cells was performed as described previously (*28*). In brief, tumor sections or cells were fixed with 4% PFA for ten minutes, washed three times with cold PBS for five minutes each, permeabilized with 0.5 % (v/v) triton X-100 (Bio-Rad, 1610407) for ten minutes, and blocked with 3% (w/v) bovine serum albumin (Sigma-Aldrich, A7906) in PBS for one hour at room temperature. Antigen retrieval was performed by incubating the sections in boiled antigen retrieval buffer

(Vector Laboratories, H-3300) for 15 minutes. Primary antibodies were added to the sections or cells and incubated overnight at 4ºC. Primary antibodies used for immunofluorescence in this study were diluted as described below: anti-BACE1 (Abcam, ab2077, RRID: AB_302817, 1:50; Thermo Fisher Scientific, MA1-177, RRID: AB_2608440, 1:50) and anti-$EGFR^{Y1068}$ (Abcam, ab40815, RRID: AB_732110, 1:100). After the incubation of the primary antibodies, the sections or cells were washed three times with cold PBS for five minutes each and then incubated with the secondary antibodies for one hour at room temperature. The secondary antibodies used in this study included Alexa Fluor 488 Donkey Anti-Mouse IgG (Invitrogen, A-21202, RRID: AB_141607, 1:200), Alexa Fluor 488 Donkey Anti-Rabbit IgG (Invitrogen, A-21206, RRID: AB_2535792, 1:200), Alexa Fluor 488 Donkey Anti-Goat IgG (Invitrogen, A-11055, RRID: AB_2534102, 1:200), and Alexa Fluor 488 Goat Anti-Rabbit (Invitrogen, A-11008, RRID: AB_143165, 1:200). For immunohistochemistry studies, horseradish peroxidase (HRP)-conjugated secondary antibodies to rabbit (Abcam, ab214880, RRID: AB_3106917) and mouse (Abcam, ab214879, RRID: AB_3678671) were utilized according to the manufacturer's instructions. After washing three times with cold PBS for five minutes each, the sections or cells were counterstained by DAPI (Cell Signaling, 4083, 1:5000) and sealed with mounting medium (Sigma-Aldrich, F4680). Finally, images were captured by a fluorescence microscope (Leica DM4000) and further analyzed with ImageJ software (https://imagej.nih.gov/). The scores of BACE1 expression were based on $BACE1^{+}$ cells in the samples: < 25% = 0; 25% - 50% = 1+; 50% -75% = 2+; and > 75% = 3+. The landmark time for Kaplan-Meier curves was discovery of the brain metastases.

BACE1 expression in the TRACERx 421 cohort

RSEM (v.1.3.3) (*46*) was used with default parameters to quantify gene expression on the full cohort of TRACERx 421 RNA-seq samples (*47*). Subsequently, only samples from LUAD tumors were considered. Expression of BACE1 was summarized across all available samples of a given tumor to calculate a median TPM (transcripts per million) per tumor. LUAD tumors were split into those with a clonal EGFR driver mutation, those with a clonal KRAS driver mutation, and those with neither based on the driver annotation described previously (*48*). In brief, mutations in KRAS or EGFR were classified as driver mutations, if there were ≥3 exact matches of the specific variant in the COSMIC cancer gene census (v.75) (49).

Sphere formation assay

Ten thousand lung cancer cells were plated per well of a 12-well plate and maintained in stem cell medium. For studies involving drug treatment, MK-8931 (30 μM) or DMSO was added to cells and incubated for four days. After seven days, tumor spheres were imaged by EVOS FL microscope (AMG). The sizes and numbers of spheres in the control and MK-8931 groups were further analyzed with ImageJ.

Cell viability assay

Cell viability assay was performed by using a Cell Titer-Glo Luminescent Cell Viability Assay Kit according to the manufacturer's instruction (Promega, G7571). For this assay, 1,000 cells were seeded per well of a 96-well plate in 100 μl of stem cell medium. Then, MK-8931, Osimertinib or DMSO (control) was added to cells. At indicated days, 50 μl of

the Cell-Titer Glo reagent was added to each well and incubated for 15 minutes. Luminescence was measured using the VICTOR Multilabel Plate Reader (Perkin Elmer). For synergy experiments, cells were treated with the indicated concentrations of MK-8931 and Osimertinib for 72 hours. Viability data was input into SynergyFinder 3.0 (*50*) to determine whether the two drugs were synergistic (score greater than 10), additive (score between -10 and 10) or antagonistic (less than -10). Scores were calculated using the BLISS synergy model.

Spheroid invasion assay

2000 cells were seeded per well in ultra-low attachment U-bottom 96 well plates in 100 μL NCC, spun for 10 minutes at 1200 rpm and allowed to form spheres for 72 hours. Plates were placed in an Incucyte and imaged every eight hours over the course of the 72 hours to confirm sphere formation. Following the 72 hour incubation, Matrigel was added in 100 μL to achieve a final concentration of 17.5% (CRUK0733-XCL-GLD) or 50% ($H1299^{GFP\text{-}Luc}$). Plates were incubated at 37°C for 30 minutes and then placed in the Incucyte for 10 days. Plates were imaged every eight hours for 10 days to track invasion of cells into the surrounding Matrigel. Changes in sphere size and invasion area were calculated using the Incucyte spheroid analysis software module or in ImageJ.

Transwell migration assay

CRUK0748-XCL cells (10^5 per insert) were seeded on polycarbonate inserts with 8.0 mm membrane pores in 24-well plates (Costar #3422) and maintained in DF12 medium for 48 hours. Cells were washed with cold PBS and those unmigrated cells (on the top of the

insert) were completely removed gently using a cotton swab. The migrated cells (on the underside of the insert) were fixed with cold methanol and stained with crystal violet. After washing away the dye, inserts were dried out and mounted on the glass slides. Images were captured with a Leica DMIRB microscope and the density of migrated cells was analyzed in ImageJ.

$A\beta_{1-42}$ enzyme-linked immunosorbent assay (ELISA)

Conditioned media was recovered from CRUK0748-XCL-GLD WT and BACE1-activated (Act #1 and Act#2) cells. Media was spun at 300 x g for 10 minutes to remove cellular debris. $A\beta_{1-42}$ concentrations were measured using the Human Amyloid beta (aa1-42) Quantikine ELISA kit (R&D Systems, DAB142) according to manufacturer's instructions. Briefly, conditioned media was diluted 1:1 with dilution buffer prior to assaying $A\beta_{1-42}$ concentrations. Absorbance was measured in a FLUOstar Omega (BMG Labtech) UV/vis spectrophotometer, and $A\beta_{1-42}$ concentrations were determined from interpolating from the standard curve.

Phospho-kinase array

Lysates from BACE1^{KO} cells were incubated with membranes from the human Proteome Profiler™ phospho-kinase array kit (R&D Systems, ARY003B) according to the manufacturer's instructions. Membranes were developed with enhanced chemiluminescence and imaged on a Chemi-Doc. Signal intensity for duplicate spots was calculated using ImageLab software and comparisons in intensity per condition made in GraphPad Prism.

Immunoblot analysis

Immunoblot analysis was performed as previously described (*28*). Briefly, cells were lysed with RIPA buffer [50 mM TrisHCl (pH 7.4), 150 mM NaCl, 2 mM EDTA, 1% (v/v) NP-40, 0.1% (w/v) SDS, protease inhibitor (one tablet per 10 mL of RIPA buffer, Roche) and phosphatase inhibitor (one tablet per 10 mL RIPA buffer, Roche) for 20 minutes on ice. Cell lysates or conditioned medium were collected and subjected to SDS-PAGE and blotted onto PVDF membranes (ASI, XR730). After blocking with 5% (w/v) non-fat milk (RPI, M17200) in Tris-buffered saline [25 mM Tris (pH 7.5) containing150 mM NaCl and 2 mM KCl] containing 0.05% Tween-20 (TBST), the membranes were incubated with primary antibodies overnight at 4°C. The following primary antibodies were used in this study: anti-BACE1 (Santa Cruz, sc-33711, RRID: AB_626716, 1:500), anti-EGFR (Bethyl Laboratories, A300-388AM, RRID: AB_386099, 1:1000; Santa Cruz, sc-365829, RRID: AB_10844017, 1:500), anti-EGFR^{Y1068} (Cell Signaling, 2234, RRID: AB_331701, 1:1000), anti-ERK1/2 (BioLegend, 686902, RRID: AB_2629535, 1:1000), anti-ERK1/2$^{T202/Y204}$ (Cell Signaling, 9106, RRID: AB_331768, 1:1000), anti-cJun (Cell Signaling, 2315, RRID: AB_490780, 1:1000), anti-cJun^{S63} (Cell Signaling, 2361, RRID: AB_490908, 1:1000), anti-MEK1 (Cell Signaling, 2352, AB_10693788, 1:1000), anti-MEK1/2$^{S217/221}$ (Cell Signaling, 9154, RRID: AB_2138017, 1:1000), and anti-GAPDH (Cell Signaling, 2118, RRID: AB_561053, 1:3000). After incubation with primary antibodies, the membranes were washed three times with TBST for ten minutes each. Membranes were then incubated with horseradish peroxidase (HRP)-linked secondary antibodies in 5% milk for one hour at room temperature. Species specific HRP-linked secondary antibodies used were anti-mouse IgG (Cell Signaling, 7076, RRID: AB_330924, 1:5000), anti-rabbit

IgG (Cell Signaling, 7074, RRID: AB_2099233, 1:5000), and anti-goat IgG (Santa Cruz, sc-2354, RRID: AB_628490, 1:5000). After washing three times with TBST for ten minutes each, signals on the membranes were developed using enhanced chemiluminescence (Advansta, K-12045) and images were acquired by a molecular imager (Bio-Rad, Universal Hood II) and analyzed by the Image Lab software (Bio-Rad). Uncropped immunoblots are shown in data file S6.

Proximity ligation assays (PLA)

MH1002 and HEK293T cells were seeded onto poly-L-ornithine and laminin coated coverslips or directly onto plastic coverslips, respectively. Cells were fixed, permeabilized, blocked, and incubated with primary antibodies against BACE1 and EGFR overnight as described in the immunofluorescence section above. Coverslips were washed and subjected to proximity ligation assay using the Duolink In Situ Red Mouse/Rabbit kit (Sigma, DUO92101) as described by the manufacturer. Cells were mounted in aqueous mounting media containing DAPI along with Alexa Fluor 488 labelled concanavalin A (ConA) (Thermo Fisher Scientific, C11252) at 100 μg/mL and imaged on a Nikon A1R inverted confocal microscope equipped with a Hamamatsu Orca Flash 4.0 V3 sCMOS with 82% High Quantum Efficiency at the Centre for Advanced Light Microscopy (McMaster University). Quantitative analysis of PLA foci per cell was performed using ImageJ (v2.1.0). A Difference of Gaussians approach was applied using a Sigma value of 4 to reduce background noise (*51*). Binary 16-bit PLA images were then created using a minimum intensity threshold of 20 and foci were counted (size set to 0.03-infinity, roundness set to 0.50-1.00). Nuclei were counted manually.

Images of foci were enhanced with minimum and maximum display values of 27 to 188 for MH1002 samples, and 1 to 130 for HEK293 samples. For MH1002 samples, DAPI and ConA images were additionally enhanced with display values of 1 to 60 and 30 to 270, respectively.

In vitro cleavage of EGFR

Recombinant catalytic domain of BACE1 (rBACE1) was purchased from R&D Systems (931-AS-050). Recombinant ectodomain of EGFR (rEGFR) was purchased from Sino Biological (10001-H08H). The two proteins were incubated together in 0.1 M sodium acetate buffer, pH 4.0 or pH 7.0, overnight at 37 °C. 0.25 µg of rEGFR was combined with 0.5, 2, or 4 µg of rBACE1 corresponding to a molar ratio of 1:1, 5:1, or 10:1 rBACE1:rEGFR. Reactions were stopped by the addition of 4X LDS sample buffer (Thermo Fisher, NP0007). To resolve cleavage products, reactions were electrophoresed on a 4-12% bis-tris gradient gel alongside BLUeye prestained protein ladder (FroggaBio, PM007-0500) and then silver stained (Pierce Silver Stain kit, Thermo Fisher, 24612) according the manufacturer's recommendations.

Amino-terminal oriented mass spectrometry of substrates (ATOMS) analysis of EGFR cleavage by BACE1

ATOMS employs isotopic labeling and quantitative tandem mass spectrometry to identify proteolytic cleavage sites (*37*). Isotopic labeling was carried out as previously described (*37*). 2 µg of protein, either BACE1 digested EGFR (in a protease to substrate ratio of 16:1 for 24 hours) or EGFR alone, were reduced using a final concentration of 25 mM

dithiothreitol (DTT) (Gold Biotechnology) in 200 mM HEPES at 37 °C for 1 hour. Samples were then alkylated to a final concentration of 60 mM iodoacetamide (IAA) (GE Healthcare) for 20 minutes in the dark at room temperature, followed by a quenching reaction to a final concentration of 40 mM DTT for 25 minutes at room temperature. The generated N-termini and lysines were isotopically labeled: the BACE-1 and EGFR sample was isotopically labeled with a final concentration of 20 mM deuterated heavy formaldehyde (13CD2O) (Cambridge Isotope Laboratories) and EGFR without BACE-1 sample was labeled with a final concentration of 20 mM light formaldehyde (12CD2O) (VWR Chemicals) with the addition of 40 mM sodium cyanoborohydride (Sigma-Aldrich). The pH was then adjusted to 6.5 and incubated at 37 °C overnight. All of the sample was loaded on a 10% polyacrylamide protein gel and ran for 30 minutes at 60 volts. The gel was then stained by addition of 50 mL of 0.1 % Coomassie Brilliant Blue (VWR m140-10g) in a 50% methanol, 10% acetic acid solution and incubated at room temp for 30 minutes on a rocker. The gel was then destained by 3 washes for 10 minutes each in a 50% methanol, 10% acetic acid solutions. The gel was imaged and the band containing all of the proteins was extracted by razor blade and stored in distilled water overnight at 4 °C. The gel was minced into approximately 1 mm cubed slices. Excess Coomassie Brilliant Blue stain was removed by 3 washes in a 50% acetonitrile, 50 mM ammonium bicarbonate solution with gentle shaking for 15 minutes. The gel was then dehydrated with 3 washes of 100% acetonitrile washes for 15 minutes. The gel was then rehydrated in 20 μL of a 10% acetonitrile, 40 mM ammonium bicarbonate buffer containing 0.02 μg/μL trypsin (Thermo Fisher, #90051) for 2 hours at 37 °C, followed by addition of 30 μL of trypsin solution before an overnight digestion. Peptides were extracted from the gel by

the addition of an extraction solution containing 60% acetonitrile, 1% Trifluoroacetic acid. The gel was washed 3 times in extraction solution with gentle shaking at room temperature for 10 minutes. After each incubation, the solutions were collected and pooled into a fresh low protein binding tube. The extraction solution was removed by lyophilization in a Savant RT 100 speed vac. The peptides were resuspended in 0.1% Trifluoroacetic acid (TFA) with gentle shaking for 30 minutes at room temperature. The sample was then subjected to c18 clean up by Sep-Pak solid phase extraction cartridges (Waters). Sep-Pak columns were conditioned with 1 x 3mL 90% methanol/0.1% TFA and washed with TFA, 1 x 2mL 0.1% TFA acid. Each sample was loaded onto a column and washed with 1x 3mL 0.1% TFA/5% methanol. Peptides were eluted from the column with 1 x 1mL 50% ACN/0.1% formic acid and lyophilized and submitted for liquid chromatography (LC)-tandem mass spectrometry (MS/MS) analysis to the Southern Alberta Mass Spectrometry core facility, University of Calgary. The LC-MS/MS data were analyzed using the database search MaxQuant software package v.2.5.2.0 at a peptide-spectrum match false discovery rate (FDR) of <0.05. Experimental spectra were compared to a Targeted FASTA reference containing only the EGFR and BACE1 sequences obtained from Uniprot. Search parameters were specified for dimethylation of the N-termini and lysines as a label. This key feature ensures that fully tryptic peptides, which canonically lack a dimethylated N-terminus are ignored by the search engine, leading to data enriched for protease generated peptides labeled both at their N-terminus and lysines if present.

BACE1 in vitro fluorescence resonance energy transfer (FRET) activity assay

Recombinant BACE1 was incubated with amyloid precursor protein peptide with the Swedish mutation in the BACE1 cleavage site or with EGFR peptide labelled with FRET donor:acceptor pairs methyl coumarin (MCA) and dinitrophenol (Dnp). APP [(MCA)SEVNLDAEFRK(Dnp)RR] was purchased from R&D Systems (ES004). EGFR peptide containing the L119 BACE1 cleavage site [(MCA)NSYALAVLSN(Lys(Dnp))RR] corresponding to amino acids 115 to 124 of EGFR, EGFR peptide containing the T130 BACE1 cleavage site [(MCA)DANKTGLKEL(Lys(Dnp))RR], and the EGFR peptide containing the G146 cleavage site [(MCA)EILHGAVRFS(Lys(Dnp))RR], all with two terminal arginines added to improve solubility, were purchased from GenScript. 0.1 µM BACE1 was incubated with 1 µM peptide for 30 minutes or 24 hours at 37 °C in 0.1 M sodium acetate buffer, pH 4.0. Liberation of fluorescence at 420 nm was measured in a Biotek Neo (Agilent) every 60 seconds for 30 minutes and then again after 24 hours. Parallel reactions were run in the presence of 1 µM MK-8931 to confirm any liberation of fluorescence was due to BACE1 activity.

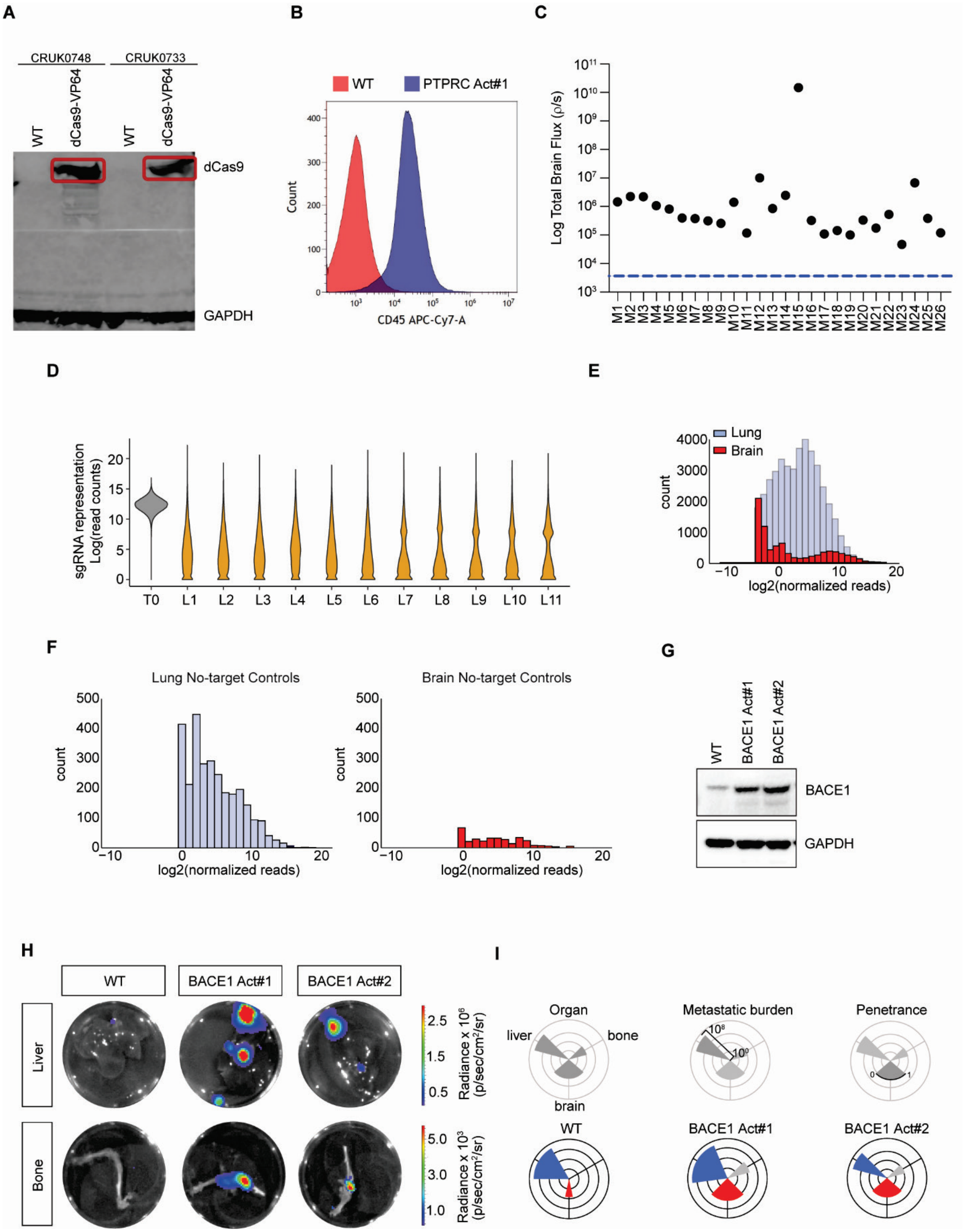
A
CRUK0748
CRUK0733
WT
dCas9-VP64
WT
dCas9-VP64
dCas9
GAPDH
B
WT
PTPRC Act#1
Count
CD45 APC-Cy7-A
C
Log Total Brain Flux (ρ/s)
M1 M2 M3 M4 M5 M6 M7 M8 M9 M10 M11 M12 M13 M14 M15 M16 M17 M18 M19 M20 M21 M22 M23 M24 M25 M26
D
sgRNA representation Log(read counts)
T0 L1 L2 L3 L4 L5 L6 L7 L8 L9 L10 L11
E
Lung
Brain
count
log2(normalized reads)
F
Lung No-target Controls
Brain No-target Controls
count
log2(normalized reads)
G
WT
BACE1 Act#1
BACE1 Act#2
BACE1
GAPDH
H
WT
BACE1 Act#1
BACE1 Act#2
Liver
Bone
Radiance x 10^6 (p/sec/cm²/sr)
Radiance x 10^3 (p/sec/cm²/sr)
I
Organ
liver
bone
brain
Metastatic burden
Penetrance
WT
BACE1 Act#1
BACE1 Act#2

Fig. S1. In vivo CRISPR activation screen identifies *BACE1* drives LUAD brain metastasis. (**A**) Immunoblot analysis of dCas9 and GAPDH expression in the indicated cell lines. CRUK0748, CRUK0748-XCL cells; CRUK0733, CRUK0733-XCL cells. (**B**) Flow cytometry analysis for activation of CD45 expression in CRUK0748-XCL-GLD (WT) or CRUK0748-XCL-GLD cells transduced with sgRNA targeting *PTPRC* (PTPRC Act#1). (**C**) Bioluminescence values following ex vivo imaging of the brains of the mice in the screen at endpoint. Blue line, baseline bioluminescent signal from the brains of naïve mice. (**D**) Violin plot of the sgRNA representation across each of the lungs (L1 to L11) analyzed from the screen relative to the T_0 cell inoculum sample. (**E**) Lung (blue) and brain (red) sgRNA distribution plots. (**F**) Distribution plots of the control sgRNAs in the lung (left) and brain (right). (**G**) Immunoblot analysis of BACE1 expression in the indicated CRUK0748-XCL-GLD cell lines following expression of *BACE1*-activating sgRNAs. GAPDH was used as a loading control. (**H**) Representative images of livers (top row) or bones (bottom row) from mice bearing orthotopic tumors from CRUK0748-XCL-GLD (WT) or BACE1-activated (BACE1 Act#1, Act#2) cells. Heat maps depict radiance values for each organ. (**I**) Petal plots summarizing the metastatic tropism of the CRUK0748-XCL-GLD (WT) or BACE1-activated (BACE1 Act#1, Act#2) cell lines implanted orthotopically in (F). Petal height indicates average total bioluminescent flux (0 to 10^8) for the group for that organ. Petal width (0 to 1) depicts the metastatic penetrance for the group (n=8 per group, N=2). GAPDH was used as the loading control in (A) and (G).

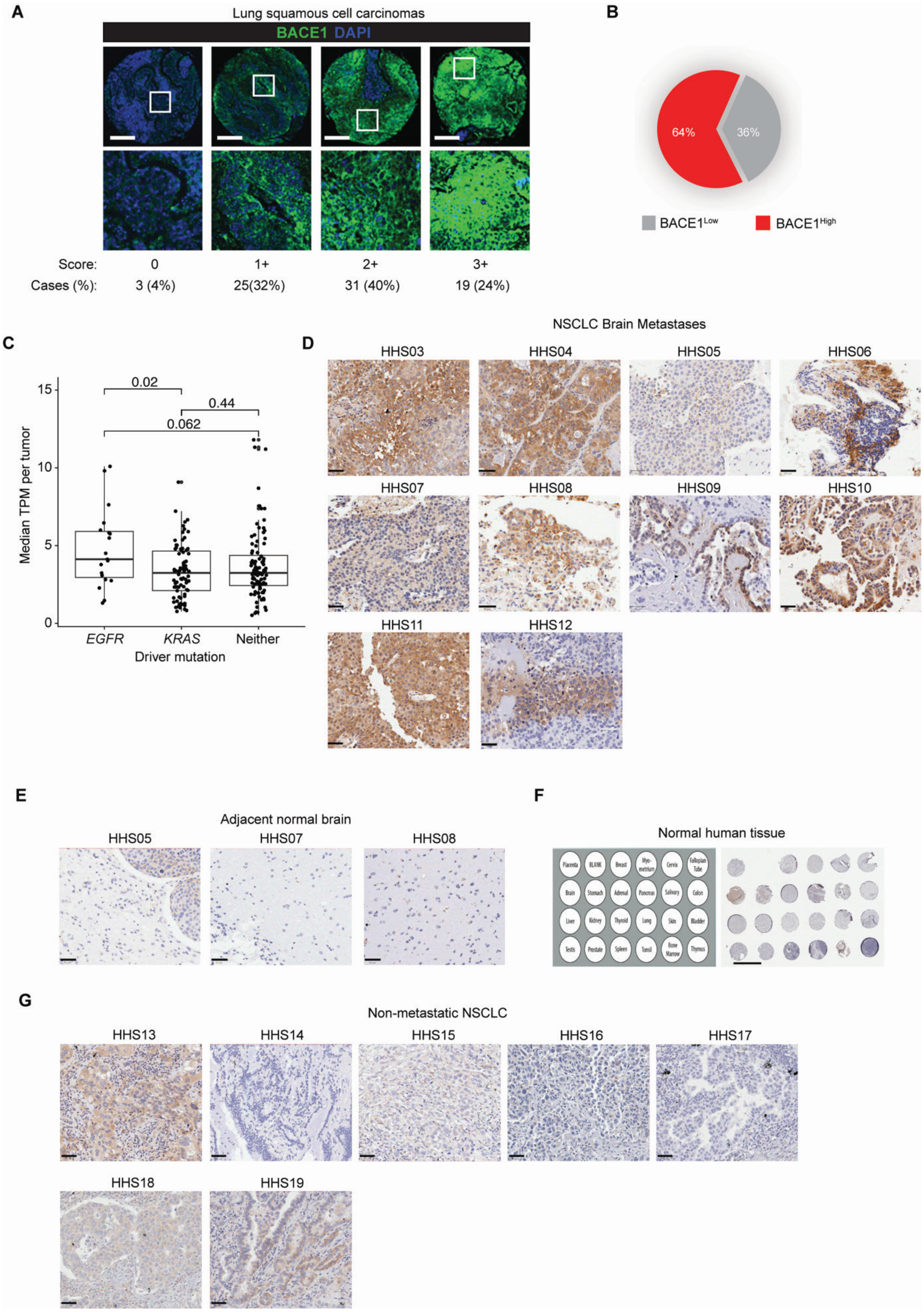
A
Lung squamous cell carcinomas
BACE1 DAPI
Score:
0
1+
2+
3+
Cases (%):
3 (4%)
25(32%)
31 (40%)
19 (24%)
B
64%
36%
BACE1Low
BACE1High
C
Median TPM per tumor
15
10
5
0
0.02
0.44
0.062
EGFR
KRAS
Neither
Driver mutation
NSCLC Brain Metastases
D
HHS03
HHS04
HHS05
HHS06
HHS07
HHS08
HHS09
HHS10
HHS11
HHS12
E
Adjacent normal brain
HHS05
HHS07
HHS08
F
Normal human tissue
Placenta
BLANK
Breast
Myo-metrium
Cervix
Fallopian Tube
Brain
Stomach
Adrenal
Pancreas
Salivary
Colon
Liver
Kidney
Thyroid
Lung
Skin
Bladder
Testis
Prostate
Spleen
Tonsil
Bone Marrow
Thymus
G
Non-metastatic NSCLC
HHS13
HHS14
HHS15
HHS16
HHS17
HHS18
HHS19

Fig. S2. BACE1 is expressed in LUAD brain metastasis and is associated with worse prognosis. (**A**) Immunofluorescent staining of BACE1 (green) in squamous cell lung carcinomas in a primary non-small cell lung cancer (NSCLC) tissue microarray (TMA). Nuclei are counterstained with DAPI (Blue). Scale bar = 250 μm. (**B**) Pie chart depicting the frequency of tumor cores according to BACE1 expression. (**C**) Normalized *BACE1* expression in the LUAD cases of the TRACERx 421 cohort associated with *EGFR* or *KRAS* clonal mutations. (**D**) Immunohistochemical staining of BACE1 in NSCLC brain metastases from Hamilton Health Sciences. Scale bar = 50 μm. (**E**) Immunohistochemical staining of BACE1 in adjacent normal brain regions from the indicated patients with NSCLC brain metastases. Scale bar = 50 μm. (**F**) Immunohistochemical staining of BACE1 in a normal human TMA. Scale bar = 3 mm. (**G**) Immunohistochemical staining of BACE1 in non-metastatic human NSCLC tumors. Scale bar = 50 μm. The data in (C) were analyzed by a two-sided unpaired Wilcoxon test.

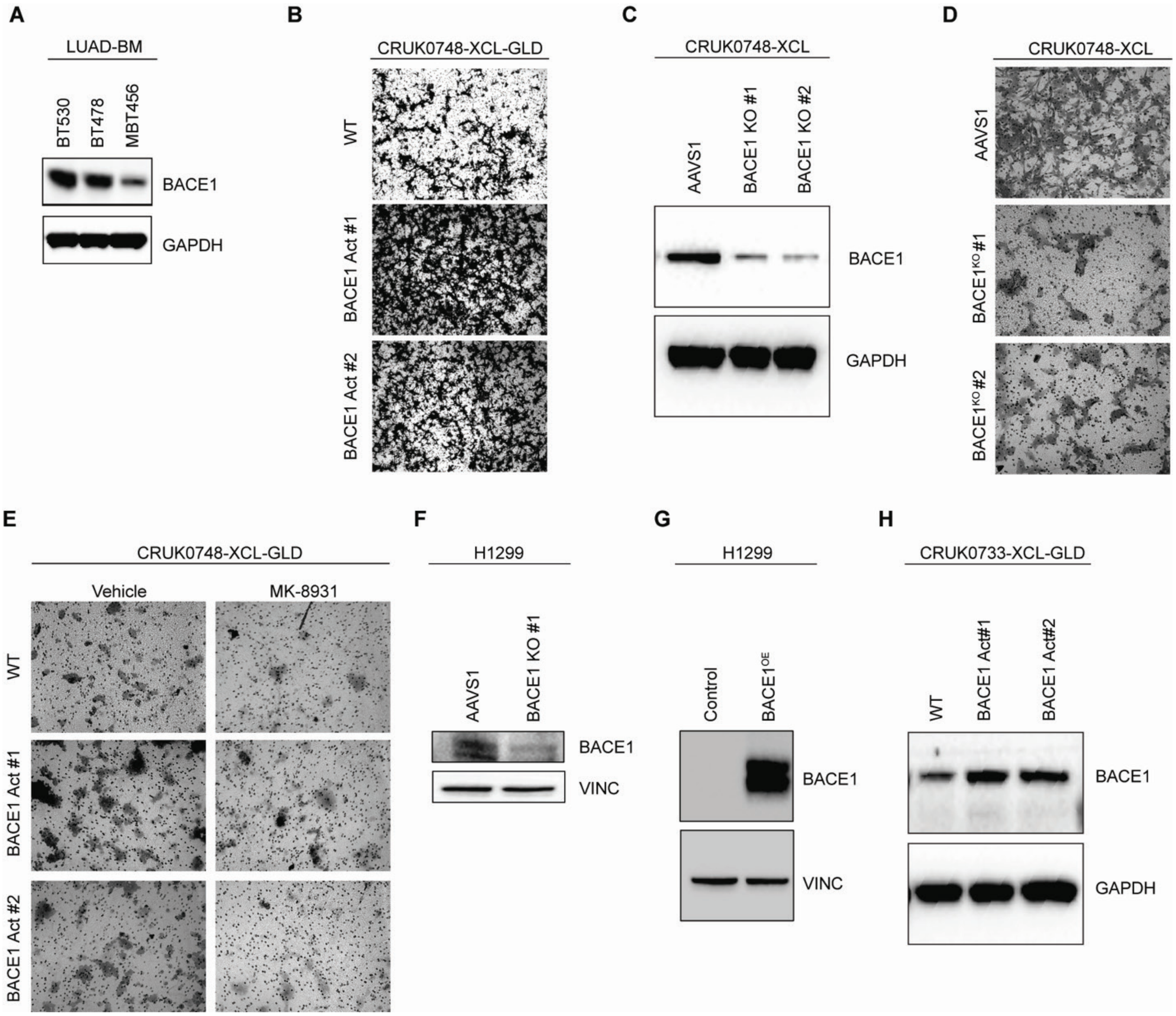

Fig. S3. BACE1 increases the migratory and invasive capacity of primary LUAD cells. (**A**) Immunoblot analysis of BACE1 and GAPDH expression in a panel of lung cancer brain metastasis (LUAD-BM) initiating cells (*N*=2). (**B**) Representative micrographs of crystal violet stained cells depicting transwell migration of CRUK0748-XCL-GLD cells following BACE1 activation. Migration proceeded for 48 hours after the cells were seeded (*n*=6, *N*=3). (**C**) Immunoblot analysis of the indicated lysates from CRUK0748-XCL BACE1^{KO} cells for BACE1 and GAPDH expression. (**D**) Representative micrographs of crystal violet stained cells depicting transwell migration of *BACE1*^{KO} CRUK0748-XCL cells. Migration proceeded for 48 hours after the cells were seeded (*n*=4, *N*=3). (**E**) Representative micrographs of crystal violet stained cells depicting transwell migration of MK-8931 (10 μM) treated CRUK0748-XCL-GLD cells. Migration proceeded for 48 hours after the cells were seeded (*n*=6, *N*=3). (**F**) Immunoblot analysis of H1299$^{GFP\text{-}Luc}$ BACE1^{KO} cells for BACE1 and Vinculin expression (*N*=2). (**G**) Immunoblot analysis of H1299$^{GFP\text{-}Luc}$ cells over-expressing BACE1 for BACE1 and Vinculin expression (*N*=2). (**H**)

Immunoblot analysis of the indicated lysates from CRUK0733-XCL-GLD cells for BACE1 and GAPDH expression following activation of BACE1 (*N*=2).

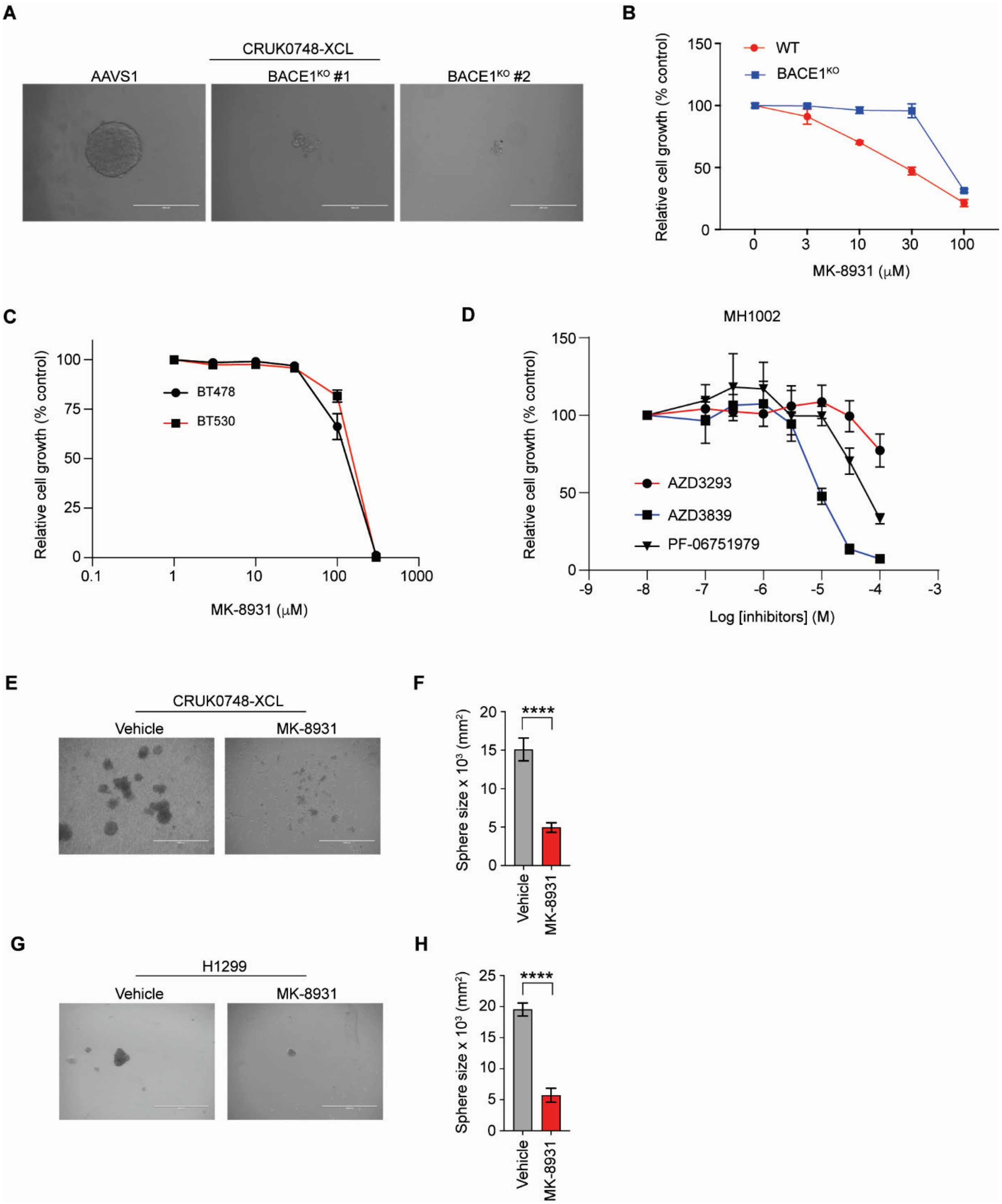

Fig. S4. BACE1 is required for the proliferation and self-renewal capacity of LUAD brain metastases. (**A**) Micrographs of CRUK0748-XCL WT and BACE1^{KO} cells grown as spheres in stem cell enriching conditions for 7 days (*n*=6, *N*=2). Scale bar = 400 μm. (**B**) Growth of wild type and BACE1^{KO} MH1002 cells following treatment with the indicated concentrations of MK-8931 for 72 h (*n*=3, *N*=2). (**C**) Growth of lung-to-brain metastasis

BMIC lines BT530 and BT478 following treatment with the indicated concentrations of MK-8931 for 72 hours (*n*=3, *N*=2). (**D**) Growth of MH1002 cells following treatment with the indicated BACE1 inhibitors for 72 hours (*n*=3, *N*=2). (**E**) Micrographs of CRUK0748-XCL cells in stem cell enriching conditions treated with 30 μM MK-8931 for 96 hours. Spheres were allowed to form for 7 days. Scale bar = 1000 μm. (**F**) Quantification of sphere size from cells imaged in (E) (*n*=6, *N*=3). (**G**) Micrographs of H1299 cells in stem cell enriching conditions treated with 30 μM MK-8931 for 96 hours. Spheres were allowed to form for 7 days. Scale bar = 1000 μm. (**H**) Quantification of sphere size from cells imaged in (G) (*n*=6, *N*=3). Data are presented as mean ± SEM. Data in (F) and (H) were analyzed by t test. **** *P*<0.0001.

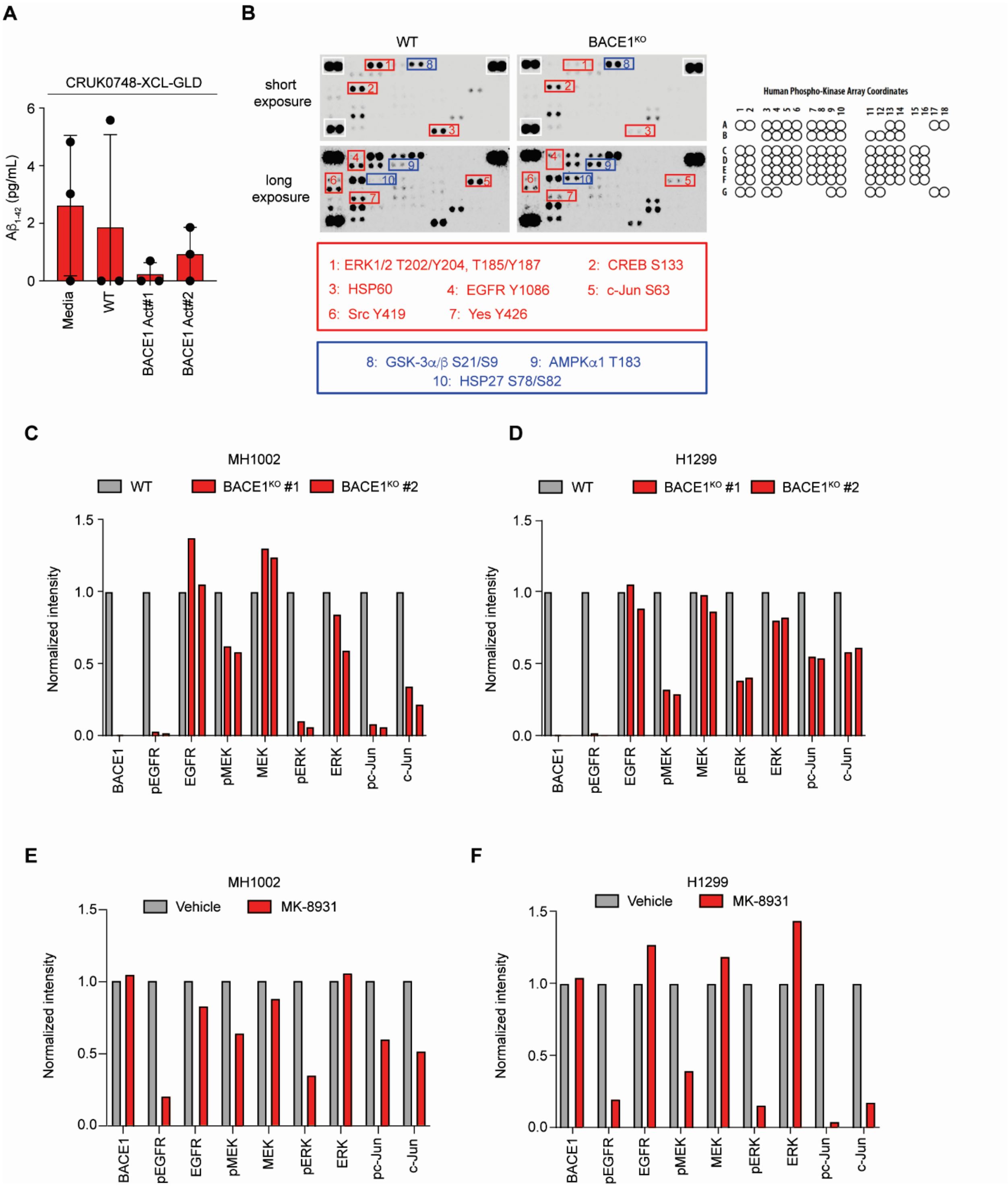

Fig. S5. BACE1 activates the EGFR/MEK/ERK axis in NSCLC. (**A**) A$\beta_{1\text{-}42}$ concentrations were measured by ELISA in the conditioned media from the indicated CRUK0748-XCL-GLD cell lines or media alone control (*n*=3, *N*=1). Individual values are shown with bars indicating the mean ± SD. (**B**) (*Left*) Human Proteome Profiler phospho-

kinase antibody array in lysates from *BACE1*^{KO} MH1002 cells highlighting additional changes in kinase activity. Red, increased; blue, decreased. (*Right*) Map of the Human Proteome Profiler phospho-kinase antibody array. (**C**) Quantification of signal intensity of the indicated proteins in lysates from MH1002 WT and BACE^{KO} cells. (**D**) Quantification of signal intensity of the indicated proteins in lysates from H1299 WT and BACE^{KO} cells. (**E**) Quantification of signal intensity of the indicated proteins in lysates from MH1002 cells treated with DMSO or 30 μm MK-8931 for 72 hours. (**F**) Quantification of signal intensity of the indicated proteins in lysates from MH1002 cells treated with DMSO or 30 μm MK-8931 for 72 hours.

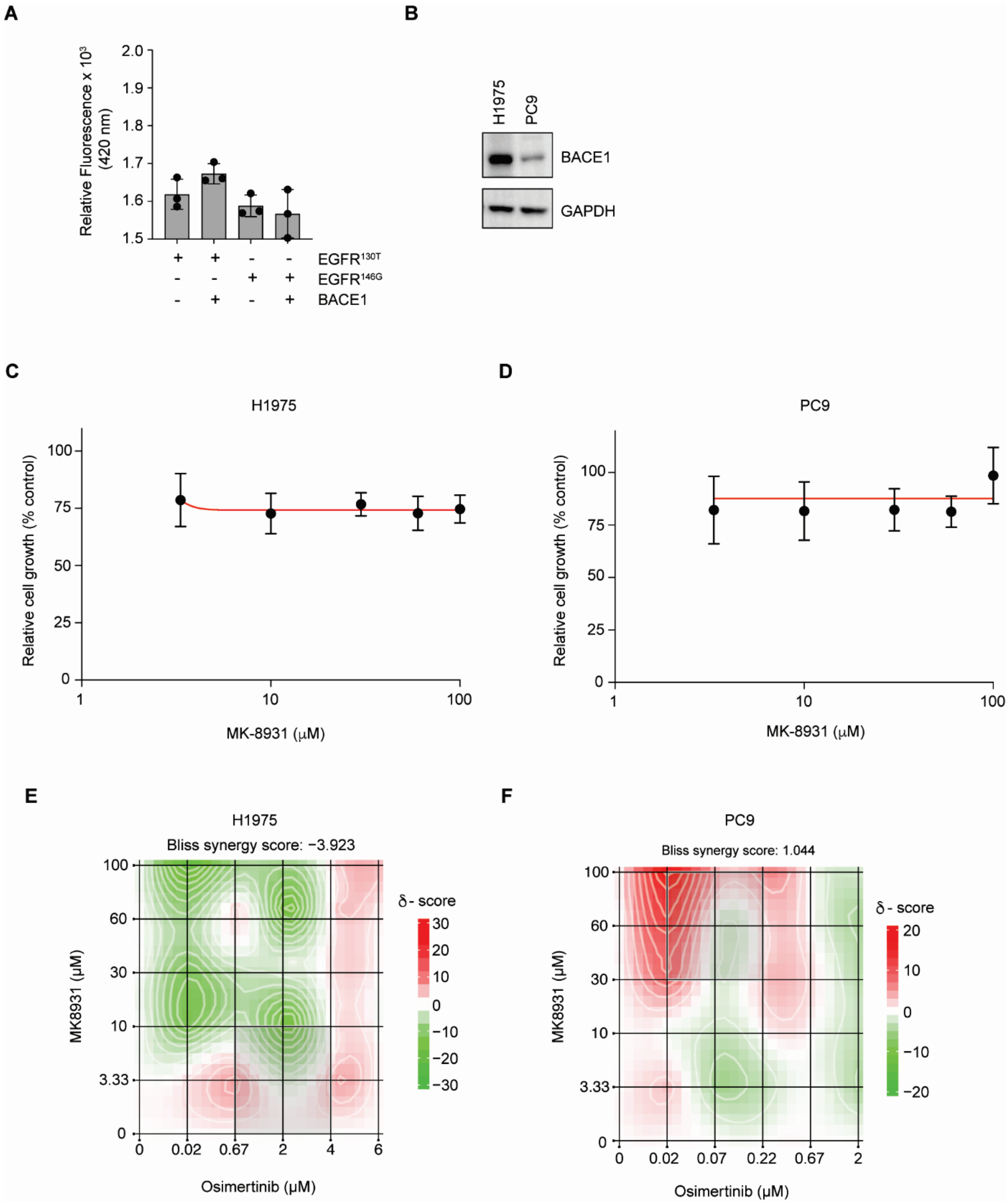

Fig. S6. EGFR is a substrate of BACE1. (**A**) BACE1 (100 nM) was incubated with EGFR peptide (1 μM) encompassing the $^{130}T{\downarrow}G^{131}$ or $^{146}G{\downarrow}A^{147}$ cleavage site labelled with methyl coumarin and dinitrophenol FRET donor:acceptor pairs. The reactions were run for 24 hours at 37 °C. Bars indicate mean fluorescence at 420 nm ± standard deviation

(*N*=2). (**B**) Immunoblot for BACE1 and GAPDH expression in the indicated EGFR mutant LUAD cell lines (*N*=2). (**C and D**) Cell viability following treatment of EGFR mutant LUAD lines H1975 (**C**) and PC9 (**D**) for 72 hours with the indicated concentrations of MK-8931 (*n*=4, *N*=3). Data are presented as mean ± SD. Red lines indicate non-linear fit of the data [log(inhibitor) vs response – variable slope (four parameters)]. (**E and F**) Topography plots displaying synergy scores of H1975 (**E**) and PC9 (**F**) determined using SynergyFinder 3.0 following treatment with the indicated concentrations of MK-8931 and Osimertinib (*45*). Synergy scores were determined using the BLISS model and BLISS scores are reported above each plot. Heatmaps indicate absolute synergy scores across the plots.

Table S1: Genetic information for patient-derived LUAD models. [a]TNM staging AJCC version 8.

Cell line	Source	Sex	Age	TNM[a] Stage	Mutations	EGFR
**CRUK0748**	Here	Male	74	3a (T2b,N2,M0)	BRCA,KRAS G12V, KEAP1	WT
**CRUK0733**	Here	Male	66	3a (T2b,N2,M0)	TP53,ARID2,FBXW7	WT
**MH1002**	Here			M1b	KRAS G12D	WT
**H1299**	ATCC	Male	43		TP53	WT
**H1975**	ATCC	Female			PIK3CA, TP53	T790M; L858R
**PC9**	ATCC	Male	45		TP53	E746_A750del

Table S2: Primer sequences utilized for sgRNA amplification for next-generation sequencing. Nucleotides shown in red are introduced to create stagger and provide diversity in the sequencing library upon multiplexing.

Primer ID	Sequence
SCp7_01	CAAGCAGAAGACGGCATACGAGATCGGTTCAAGTGACTGGAGTTCAGACGTGTGCTC<br>TT CCGATCTTCTACTATTCTTTCCCCTGCACTGT
SCp7_02	CAAGCAGAAGACGGCATACGAGATGCTGGATTGTGACTGGAGTTCAGACGTGTGCTC<br>TT CCGATCTTCTACTATTCTTTCCCCTGCACTGT
SCp7_03	CAAGCAGAAGACGGCATACGAGATTAACTCGGGTGACTGGAGTTCAGACGTGTGCTC<br>TT CCGATCTTCTACTATTCTTTCCCCTGCACTGT
SCp7_04	CAAGCAGAAGACGGCATACGAGATTAACAGTTGTGACTGGAGTTCAGACGTGTGCTCT<br>T CCGATCTTCTACTATTCTTTCCCCTGCACTGT
SCp7_05	CAAGCAGAAGACGGCATACGAGATATACTCAAGTGACTGGAGTTCAGACGTGTGCTCT<br>T CCGATCTTCTACTATTCTTTCCCCTGCACTGT
SCp7_06	CAAGCAGAAGACGGCATACGAGATGCTGAGAAGTGACTGGAGTTCAGACGTGTGCTC<br>TT CCGATCTTCTACTATTCTTTCCCCTGCACTGT
SCp7_07	CAAGCAGAAGACGGCATACGAGATATTGGAGGGTGACTGGAGTTCAGACGTGTGCTC<br>TT CCGATCTTCTACTATTCTTTCCCCTGCACTGT
SCp7_08	CAAGCAGAAGACGGCATACGAGATTAGTCTAAGTGACTGGAGTTCAGACGTGTGCTCT<br>T CCGATCTTCTACTATTCTTTCCCCTGCACTGT
SCp7_09	CAAGCAGAAGACGGCATACGAGATCGGTGACCGTGACTGGAGTTCAGACGTGTGCTC<br>TT CCGATCTTCTACTATTCTTTCCCCTGCACTGT
SCp7_10	CAAGCAGAAGACGGCATACGAGATTACAGAGGGTGACTGGAGTTCAGACGTGTGCTC<br>TT CCGATCTTCTACTATTCTTTCCCCTGCACTGT
SCp7_11	CAAGCAGAAGACGGCATACGAGATATTGTCAAGTGACTGGAGTTCAGACGTGTGCTCT<br>T CCGATCTTCTACTATTCTTTCCCCTGCACTGT
SCp7_12	CAAGCAGAAGACGGCATACGAGATTATGTCTTGTGACTGGAGTTCAGACGTGTGCTCT<br>T CCGATCTTCTACTATTCTTTCCCCTGCACTGT
SCp7_13	CAAGCAGAAGACGGCATACGAGATATTGGATTGTGACTGGAGTTCAGACGTGTGCTCT<br>T CCGATCTTCTACTATTCTTTCCCCTGCACTGT
SCp7_14	CAAGCAGAAGACGGCATACGAGATATACTCGGGTGACTGGAGTTCAGACGTGTGCTC<br>TT CCGATCTTCTACTATTCTTTCCCCTGCACTGT
SCp7_15	CAAGCAGAAGACGGCATACGAGATTATGAGAAGTGACTGGAGTTCAGACGTGTGCTCT<br>T CCGATCTTCTACTATTCTTTCCCCTGCACTGT
SCp7_16	CAAGCAGAAGACGGCATACGAGATGCACAGTTGTGACTGGAGTTCAGACGTGTGCTC<br>TT CCGATCTTCTACTATTCTTTCCCCTGCACTGT
SCp7_17	CAAGCAGAAGACGGCATACGAGATCGTGGATTGTGACTGGAGTTCAGACGTGTGCTC<br>TT CCGATCTTCTACTATTCTTTCCCCTGCACTGT
SCp7_18	CAAGCAGAAGACGGCATACGAGATTAGTAGAAGTGACTGGAGTTCAGACGTGTGCTCT<br>T CCGATCTTCTACTATTCTTTCCCCTGCACTGT
SCp7_19	CAAGCAGAAGACGGCATACGAGATGCACGATTGTGACTGGAGTTCAGACGTGTGCTC<br>TT CCGATCTTCTACTATTCTTTCCCCTGCACTGT
SCp7_20	CAAGCAGAAGACGGCATACGAGATCGGTAGCCGTGACTGGAGTTCAGACGTGTGCTC<br>TT CCGATCTTCTACTATTCTTTCCCCTGCACTGT
SCp7_21	CAAGCAGAAGACGGCATACGAGATTAGTTCTTGTGACTGGAGTTCAGACGTGTGCTCT<br>T CCGATCTTCTACTATTCTTTCCCCTGCACTGT
SCp7_22	CAAGCAGAAGACGGCATACGAGATTACAAGTTGTGACTGGAGTTCAGACGTGTGCTCT<br>T CCGATCTTCTACTATTCTTTCCCCTGCACTGT
SCp7_23	CAAGCAGAAGACGGCATACGAGATATCACTGGGTGACTGGAGTTCAGACGTGTGCTC<br>TT CCGATCTTCTACTATTCTTTCCCCTGCACTGT
SCp7_24	CAAGCAGAAGACGGCATACGAGATCGCATCAAGTGACTGGAGTTCAGACGTGTGCTC<br>TT CCGATCTTCTACTATTCTTTCCCCTGCACTGT

SCp7_25	CAAGCAGAAGACGGCATACGAGATGCACGACCGTGACTGGAGTTCAGACGTGTGCTCTT CCGATCTTCTACTATTCTTTCCCCTGCACTGT
SCp7_26	CAAGCAGAAGACGGCATACGAGATTACACTCCGTGACTGGAGTTCAGACGTGTGCTCTT CCGATCTTCTACTATTCTTTCCCCTGCACTGT
SCp7_27	CAAGCAGAAGACGGCATACGAGATCGGTCTAAGTGACTGGAGTTCAGACGTGTGCTCTT CCGATCTTCTACTATTCTTTCCCCTGCACTGT
SCp7_28	CAAGCAGAAGACGGCATACGAGATATGTTCGGGTGACTGGAGTTCAGACGTGTGCTCTT CCGATCTTCTACTATTCTTTCCCCTGCACTGT
SCp7_29	CAAGCAGAAGACGGCATACGAGATCGTGGACCGTGACTGGAGTTCAGACGTGTGCTCTT CCGATCTTCTACTATTCTTTCCCCTGCACTGT
SCp7_30	CAAGCAGAAGACGGCATACGAGATATTGAGCCGTGACTGGAGTTCAGACGTGTGCTCTT CCGATCTTCTACTATTCTTTCCCCTGCACTGT
SCp5_01	AATGATACGGCGACCACCGAGATCTACACTCTTTCCCTACACGACGCTCTTCCGATCTTTGTGGAAAGGACGAAACACCG
SCp5_02	AATGATACGGCGACCACCGAGATCTACACTCTTTCCCTACACGACGCTCTTCCGATCTCTTGTGGAAAGGACGAAACACCG
SCp5_03	AATGATACGGCGACCACCGAGATCTACACTCTTTCCCTACACGACGCTCTTCCGATCTGCTTGTGGAAAGGACGAAACACCG
SCp5_04	AATGATACGGCGACCACCGAGATCTACACTCTTTCCCTACACGACGCTCTTCCGATCTAGCTTGTGGAAAGGACGAAACACCG
SCp5_05	AATGATACGGCGACCACCGAGATCTACACTCTTTCCCTACACGACGCTCTTCCGATCTCAACTTGTGGAAAGGACGAAACACCG
SCp5_06	AATGATACGGCGACCACCGAGATCTACACTCTTTCCCTACACGACGCTCTTCCGATCTTGCACCTTGTGGAAAGGACGAAACACCG
SCp5_07	AATGATACGGCGACCACCGAGATCTACACTCTTTCCCTACACGACGCTCTTCCGATCTACGCAACTTGTGGAAAGGACGAAACACCG
SCp5_08	AATGATACGGCGACCACCGAGATCTACACTCTTTCCCTACACGACGCTCTTCCGATCTGAAGACCCTTGTGGAAAGGACGAAACACCG
pXPR502_For	GAGGGCCTATTTCCCATGATTC
pXPR502_Rev	CAAACCCAGGGCTGCCTTGGAA

Supplementary Movies

Movie S1. Invasion of CRUK0733 cells. Representative time lapse images from the Incucyte of invading CRUK0733-XCL-GLD cells.

Movie S2. Invasion of CRUK0733 cells following increased expression of BACE1. Representative time lapse images from the Incucyte of CRISPR-activated *BACE1* (BACE1-act) CRUK0733-XCL-GLD cells.

Supplementary Data Files

Data file S1. Normalized read counts from next generation sequencing of CRISPR activation screen.

Data file S2. Clinicopathological data associated with Hamilton Health Sciences and TCGA patient cohorts.

Data file S3. Proteome Profiler antibody array key.

Data file S4. BACE1 cleavage sites detected in EGFR by ATOMS.

Data file S5. Individual-level data.

Data file S6. Uncropped immunoblots.
